# The anatomy of urban social networks and its implications in the searchability problem

**DOI:** 10.1038/srep10265

**Published:** 2015-06-02

**Authors:** C. Herrera-Yagüe, C. M. Schneider, T. Couronné, Z. Smoreda, R. M. Benito, P. J. Zufiria, M. C. González

**Affiliations:** 1 Department of Civil and Environmental Engineering, Massachusetts Institute of Technology, Cambridge, MA, USA; 2 Depto. Matemática Aplicada a las Tecnologías de la Información y las Comunicaciones (TIC), ETSI Telecomunicación, Universidad Politécnica de Madrid (UPM), Spain; 3 Cátedra Orange. Universidad Politécnica de Madrid (UPM), Spain; 4 Sociology and Economics of Networks and Services department, Orange Labs, Issy les Moulineaux, France; 5 Grupo de Sistemas Complejos, Departamento de Física y Mecánica, ETSI Agrónomos, Universidad Politécnica de Madrid (UPM), Spain

## Abstract

The appearance of large geolocated communication datasets has recently increased our understanding of how social networks relate to their physical space. However, many recurrently reported properties, such as the spatial clustering of network communities, have not yet been systematically tested at different scales. In this work we analyze the social network structure of over 25 million phone users from three countries at three different scales: country, provinces and cities. We consistently find that this last urban scenario presents significant differences to common knowledge about social networks. First, the emergence of a giant component in the network seems to be controlled by whether or not the network spans over the entire urban border, almost independently of the population or geographic extension of the city. Second, urban communities are much less geographically clustered than expected. These two findings shed new light on the widely-studied searchability in self-organized networks. By exhaustive simulation of decentralized search strategies we conclude that urban networks are searchable not through geographical proximity as their country-wide counterparts, but through an homophily-driven community structure.

In the last decade social network analysis methods have allowed us to uncover local and global patterns^1^, locate influential individuals 2, and examine network dynamics^3^. The study of macro-level social networks traces the outcomes of collective and large-scale social interactions such as economic development^4^, resource transfer^5^, disease transmission^6^, and communications^7^ over a large population. In these cases, networks nodes represent individuals, and links are generally defined by friendships or acquaintances among them. Well documented structural patterns of these networks are: the positive correlations in the degree of adjacent nodes 8, a short diameter (increasing as the natural logarithm of the number of nodes)^9^, and network transitivity or clustering, which is the propensity for nodes pairs to be connected if they share a mutual neighbor^9^. Interestingly, social networks are also divided in groups or communities, and the existence of such communities alone can produce both degree correlations and high clustering^10^. On the other hand, some social links are also the consequence of similar attributes of their nodes. Similar people tend to select each other^11^,^12^, they communicate more frequently and present stronger social interactions^7^.

Parallel to the rise of social network analysis, and often using similar data sources, human mobility patterns have also considerably evolved in the recent years^13^,^14^. An interesting topic of study which has started to grow recently is to combine findings from both areas to explain the relationship between social networks and geographical space. Evidently, social contacts can exist only if there is the opportunity for such contacts to be created. This explains, for example, the ubiquitous findings showing that geographic proximity favors the existence of social contacts^15^,^16^. Additionally, Network Communities (locally dense areas of the social graph) have been analyzed in several country scale social networks when the spatial positions of the nodes are known, as more and more often is the case in social networks resulting from information and communication technologies^17^. A well-documented result of these communities is that they retrace national^18^,^19^ and administrative borders^20^ when studied at a country scale.

The spatial dispersion of social contacts at country scale has been studied in the context of transportation planning (see^21^ and references therein). Kowald *et al.* made a comparative study of social ties and their distances, from surveyed of individuals within cities in three different continents. They reported that although the ultimate models need to incorporate the characteristics of egos, ties, and transportation facilities, there is a general trend of a power law decay of social ties with distance. In this work we want to explore the group structure social networks in cities and its relation to space. The size of social groups has important implications our societies, Simmel^22^ viewed the increasing size in networks groups as the origin of the isolation of individuals. These implications and the related literature are out of the scope of this work. Social networks studies within cities have measured the role of the density of social ties^23^,^24^ or face to face encounters^25^,^26^. Here, we are interested in the analysis of communities within cities and their relation to space. Despite some analysis of communities within cities^27^,^28^, there is still lack of knowledge on a clear structure of urban social networks in space. Specifically, how connected components emerge with distance^29^ in urban social networks.

Here we assign each mobile phone user to a fixed location corresponding to his/her most commonly used zipcode or mobile phone tower with the goal of systematically studying the spatial properties of their social networks at different scales, including the formation of a giant component in space. The geographic distance between two nodes is then defined as the distance of their respective most common locations, typically home or work. It is expected that within cities this distance should not be a strong limiting factor in the creation of social ties as it may be other factors that define their social distance. Social distance is given by differences between groups of society, including differences such as socio-demographic, race or social identity^30^. Searchability is a well-established network property that relates to both geographic proximity and social distance: ordinary people are capable of directing messages only through their acquaintances and to reach any target person in only a few steps. Milgram^31^,^32^ first discovered this property, in a social experiment that routed letters across U.S. In the light of email communication, Dodds *et al.*^33^ showed that when routing a message to a target, people selected in the first steps acquaintances that could be geographically close to that target. However, in the latests steps, participants selected acquaintances that could belong to the professional group of the target (i.e., socially close). Up to now, the network structure that makes searchability possible has not been empirically measured in large-scale social networks.

We designed our study to explore the role of both social and geographic distances in social networks. Social distance is not trivially defined in social networks with data passively collected without much information about the attributes of the nodes. Introducing a metric of social distance for these cases is an interesting question, but out of the scope of this work. Watts *et al.* defined the social distance between two nodes as the difference in hierarchy levels of the two smallest groups the nodes belonged to^34^. Here, we use a similar definition, proposed by Kleinberg *et al.*^35^: social distance 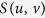 between nodes 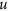 and 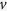 is the number of nodes in the smallest group containing 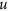 and 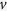. In this work we define social groups as network communities, which are locally dense sub-networks. Networks communities are thus a central aspect to the analysis of social networks, being the source of their structural properties (degree correlation and high clustering) and consequence of non-structural properties, such as homophily^36^. The detection of network communities (modules or groups) is a difficult task that has attracted much attention in the last few years^37^. Here we adopt a well-established method that detects communities by optimizing the Newman Girvan modularity metric^38^.

We first present a general description of the measured social networks, with focus on the small-world properties and link-distance distributions. Next, we report the performance of different routing strategies and show that geogreedy strategies (choosing the smallest geographical distance to the target) are ineffective within cities while strategies based on social distance (choosing within the smallest community) still work. We discover two features of urban social networks that cause the failure of geographic strategies: urban communities are geographically dispersed and there is not a large connected component in groups of nodes defined by their geographic proximity. We further measure in the urban networks how the density of links 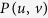 decays with increasing group size (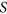) or distance. We find that the probability of finding a link between individuals 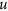 and 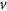 in a group of size 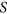 scales as 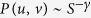, with 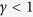 when groups (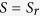) are defined by users living within geographic balls of a certain radius 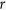. This is in contrast with observations at the national scale which report 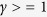
15. These results support the evidence that while geogreedy algorithms work to reach a target’s city, they fail within urban borders. In addition, we show that the condition 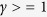 still holds when groups (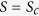 ) are defined by social distance. These results of urban groups defined by either social distance or geographic distance are in nice agreement with the analytic conditions of networks searchability^39^ and support the results reported in routing experiments^33^. This work provides novel evidence of social networks: urban networks form geographically dispersed communities that make them searchable.

## Results

### Network Structure

**Figure 1:**
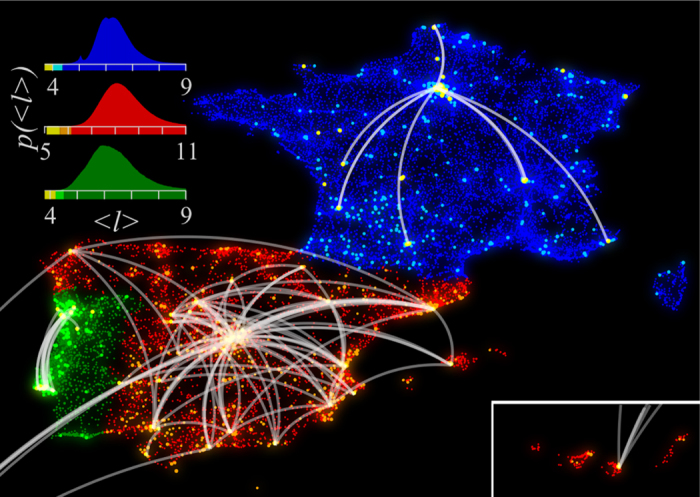
Visualization of central places in France, Spain and Portugal. Each circle represents a mobile phone tower and its color (the brighter the more central) corresponds to the inverse of closeness centrality 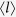 (average number of hops to any other person) of the most central people in this tower. People are always assigned either to their billing address or most used tower. White lines highlight the social network between the 50 most central persons of each country. In the three insets the distribution of the 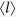 of all persons and the relation to the used color are also shown. This figure was created using Grace and Inkscape.

Our data set contains information for 7 billion mobile phone interactions gathered during a 6 month period in France, Portugal and Spain. We report the structural network characteristics in table 1. These results confirm that the networks exhibit the *small world* property, with the average number of people in the shortest path between a sender and a recipient 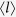 is 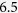, 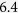, and 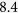 in the different countries, similar to the values reported in previous works^7^,^40^. As a sole illustration of the resulting networks, we extract the spatial distribution of the most central people in the network, considering someone is more central if he/she is in average closer to everyone else in the graph (closeness centrality). In Fig. 1 we show the distribution of the average graph distance between a sender and all possible recipients 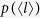 among the population for each country. This value is also known as the inverse of the closeness centrality^41^ and it ranges from 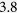 to 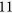, so everyone in the country is in average within 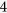 hops from the most central people and within 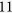 of the less central ones. Each dot represents a mobile phone tower, which is our smallest spatial resolution. In order to expose the backbone of the social network, the color intensity of each mobile phone tower represents the closeness centrality of the most central person in that tower. Additionally, the links highlight the social connections only among the 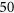 most central people in each country, showing significant differences in the social network analyzed in the three countries

**Figure 2:**
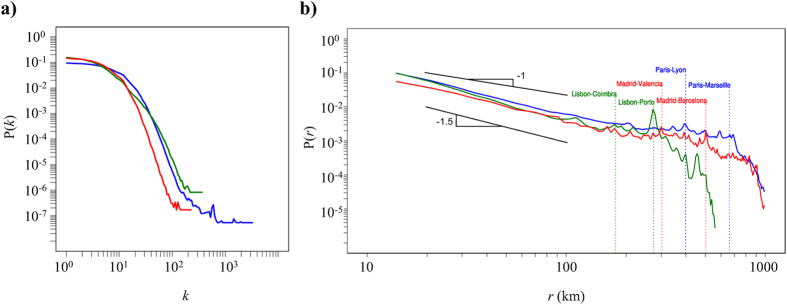
Country-wide social networks structure. (**a**) Degree distribution for each of the country level networks. (**b**) Probability of a link to have distance 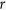 in each of the networks. Distances are grouped in 7 km bins. In all three countries, distribution present a power law decay (exponents between −1 and −1.5) up to 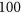 km. A large fraction of links lie within the same tower (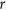 = 0), averaging 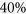 in Spain (red), 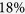 in France (blue) and 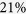 in Portugal (green).

Regarding degree distribution, our three networks present the common heavy-tail distribution found in previous works with social networks^7^,^4^. Degree distributions for all three networks are shown in Fig. 2a (details about power-law fitting can be found in Table S1). We note the existence of hubs (nodes with very high number of connections) in all three networks. In order to measure geographic proximity between individuals we need to assign a location to each of them. In our study, users are located in their billing zip code (Spain) or their most used tower (France and Portugal). Spain zip codes are geolocated according to geonames database, available at http://downloads.geonames.org/export/zip, and grouped according to latitude and longitude since some zip codes have identical coordinates. Towers coordinates were provided by the carrier. In total 8,928 different locations are available in Spain, 17,475 in France and 2,209 in Portugal. It is well documented that the probability of finding a social tie decreases with geographic proximity, regardless the proxy used to infer the social network: blogs^15^, location based social networks^43^,^44^ or mobile phone data^7^,^18^,^42^. In all of them the fraction of social links between nodes that are within distance 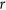 from each other decreases (at least in a certain range) as a power law, with exponents between −1 and −2. As shown in Fig. 2b, our data fits this behavior for all three networks. Kowald *et al.*^21^ present a careful analysis of the decaying function observing distance bands depending on the population, similar analysis on this data remain to further studies.

Moreover, due to the high number of links considered we are able to observe long-range peaks. The reason for these peaks is the heterogeneity in the spatial distribution of population (we observe the same peaks even if we randomize the links while keeping actors in the same location). Once established that the short paths exist all across the network, we explore the success of routing strategies at two levels: intercity and intracity.

### Exploring Routing Strategies

In order to gather insights on the social network structure, we investigate the well-known searchability condition. We explore different routing strategies on the social networks described above. We separate the routing experiment into two phases: intercity routing and intracity routing.

Intercity routing seeks to reach the correct city while intracity routing searches for the individual target within a city. Cities are defined by their administrative borders. In this study we consider two scales: provinces and municipalities as shown in Fig. S5. On both phases, we test different decentralized routing strategies which employ only information of neighbor nodes (also called contacts or friends). In a random search (**ran**), individuals route the message by randomly selecting a neighbor node that did not have had the message previously. Geographical routing (**geo**) passes the message to the contact that is geographically closest to the final target, whereas degree routing (**deg**) selects the friend with the highest number of friends. Finally, community routing (**com**) forwards the message to a friend such that he/she belongs to the smallest community containing the target (see details in the **Methods** section).

**Figure 3:**
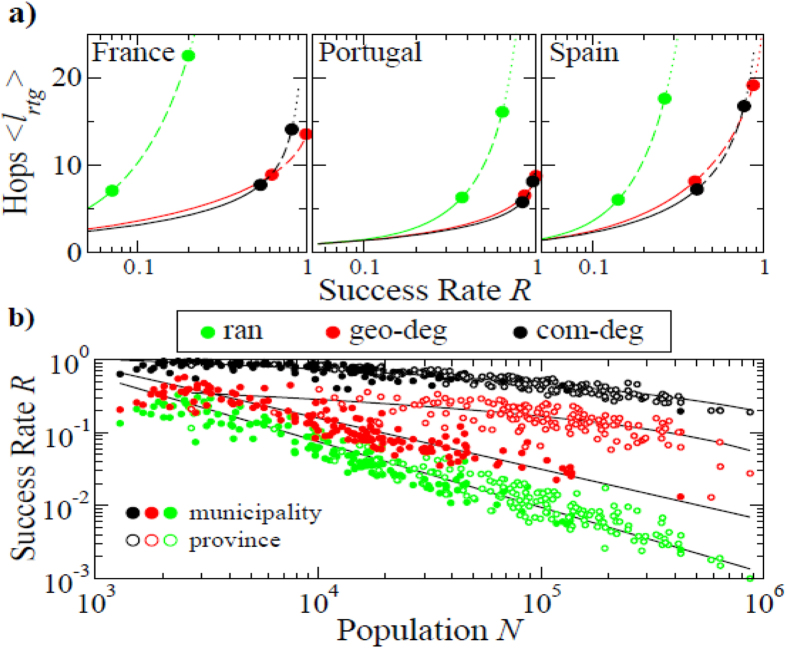
Results for different routing strategies in both stages. (**a**) Dependence of the number of hops l on the success rate 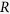 for intercity routing (results for completing the delivery within 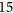 and 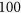 hops are highlighted by circles). (**b**) Success rate versus population size for three strategies in 155 municipalities and 150 provinces. All logarithmic and power-law functions are guides to the eye.

Our intercity simulations results presented in Fig. 3a indicate that both *geo* and *com* routing are able to reach the target cities. Moreover, the success rate depends only logarithmically on the population size of the destination city (Fig. S8), confirming that both strategies are equally efficient. The intercity experiment can be replicated in our homepage^45^. Geographic strategies had already been reported successful using a half million bloggers network across the US^15^. However, intracity routing has not been previously explored because both the low sample size of the network (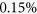 of US population) and the lack of information of the coordinates of individuals within cities obliged to relax the modeled network structure: namely, nodes were allowed to forward messages to anyone else within the target city, even if they were not directly connected. In contrast, our larger population sample (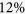
–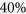) and much smaller spatial resolution (mobile phone tower scale) allow us to explore routing inside cities using strict routing among connected individuals.

Next, we explore routing strategies by analyzing the network properties within the geographic administrative borders at two scales: provinces as upper limits (usually containing large cities plus suburbs) and municipalities as lower limits (see SI for details). Thus, we analyze the three different routing strategies in 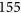 social networks from the large municipalities and all 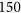 provinces of the three countries. In contrast to intercity routing, routing inside municipalities is significantly more successful if the strategy uses community information (Figs. S10–S15 show additional strategies). For different routing strategies Fig. 3b shows the success rate for municipalities (filled circles) and provinces (open circles) in each country as a function of the population size N; an upper limit of 100 hops was employed and Fig. S24 shows results with a smaller upper limit. We find that at both scales the community based routing is efficient because of the slow decay in success rate 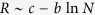 (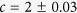 and 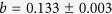) and in contrast to the random strategy, which as expected decays almost reversely linear as 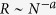 (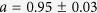). Interestingly, the geographically based routing presents a crossover behavior between municipalities (only intracity routing) and provinces (including an initial intercity stage). This behavior is due to the fact that a province consists of several municipalities. Although the geographically based routing reaches the correct municipality, within the municipality this strategy fails. This explains the different scaling observed for geographic routing in municipalities and provinces: while within municipalities the routing success rate scales similarly to the random routing 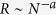 (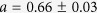), the province routing success rate scales similarly to community routing 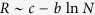 (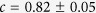 and 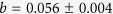), but with a lower success rate as a consequence of its inefficacy within municipalities.

In the next sections we show that the failure of the geographic routing within cities lies in two previously unknown spatial properties of urban social networks: lack of short-range connectivity and geographical dispersion of urban communities.

### Connectivity collapse within cities

**Figure 4:**
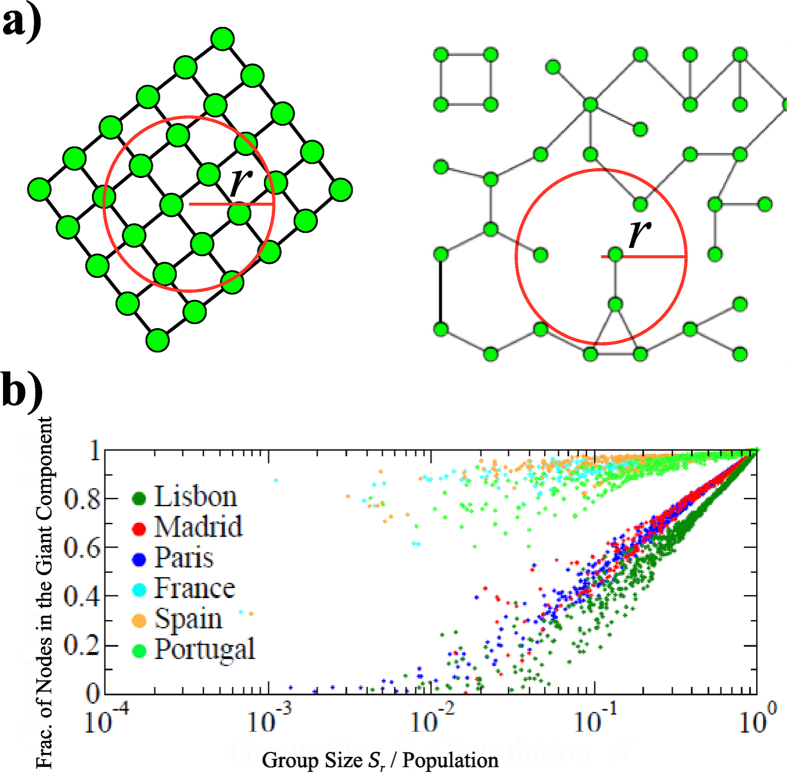
Short range connectivity (**a**) In a 2D lattice (left), any geographic ball contains a connected network, however this is not the case for any network (right) where the path between two nodes within a geographic ball might include nodes out of the ball if the network induced by the nodes within the ball is not connected. (**b**) Fraction of nodes in the giant component as a function of the relative size of the geographic ball for the three capitals compared to the country-wide networks. Each of the 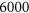 dots in the figure was calculated by selecting 2 nodes 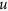 and 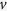 at random within a city or within the country, extracting the subnetwork defined by the ball whose center is in 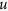 and radius up to 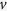, and identifying the number individuals that belonged to the giant component of such subnetwork.

A necessary condition for any geogreedy algorithm to succeed in a routing experiment is that the subgraph induced by the nodes located within any geographic ball of radius 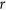 must be connected. This is equivalent to saying that if a message headed to target user B has reached a user A, A and B are in the same connected component within the subgraph induced by those nodes included in the circle whose center is in B and has radius up to A. While this is granted in a lattice our results show that is not necessarily the case in a real-world network (see Fig. 4a). We test this structure in our data using geometric and social distances. We divide the network into groups of size 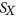 using either geographic balls (while in this work we only consider 2D geographic *circles* we keep the term *balls* for consistency with previous theoretical work^35^ which has been generalized to higher dimensions) of a certain radius 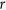 (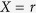) or existing communities (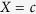) A natural question emerging then is: which is the critical radius 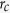 so that geographic balls with 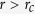 are likely to contain a connected network? Interestingly we observe that there is not a unique 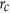, but rather this radius is defined by the size of a city, so that only geographic balls containing entire cities contain a connected network.

We illustrate this fact further by calculating the size of the largest connected component within different radius and group sizes, performing this analysis centered in different locations from the capital municipality (city) or centered in a province of the three countries. Figure 4b shows that the fraction of nodes in the giant component is much smaller within cities than within provinces. Surprisingly, we find that this lack of connectivity is not caused by not having enough short-distance links (actually between 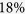 and 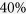 of links are within the same location (tower or zip-code)). When we zoom into a region of the city we find small highly clustered groups which form islands; the paths among these geographically neighboring groups exist through people living far away.

**Figure 5:**
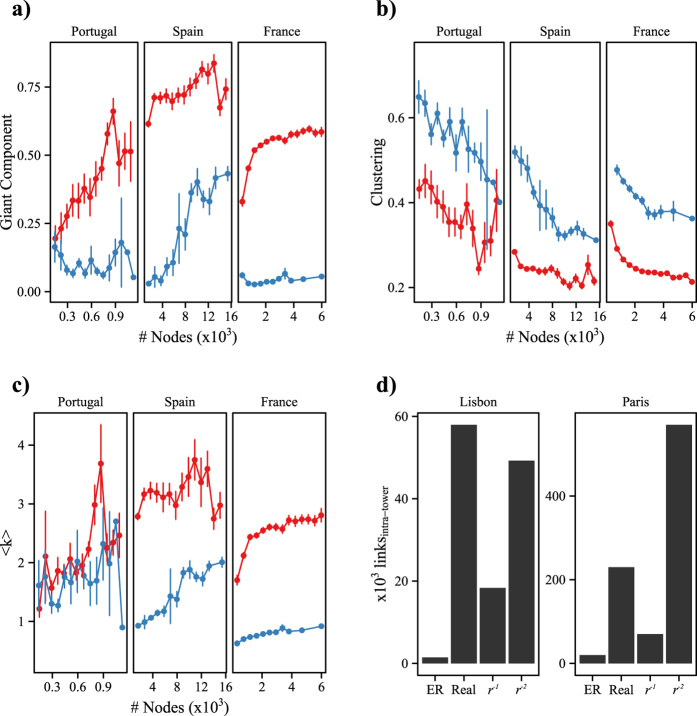
Connectivity collapse within cities. (**a**) Relation between population size and fraction of nodes in the giant component for all towers in the capital cities (blue) and municipalities in the country within the same range of population (red). Errors bars represent the standard error of the mean 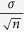. The size of the connected components within municipalities tends to be higher than within towers of the same size. (**b**) and (**c**) depict the causes of this behavior, smaller average degree and higher clustering are the reasons why the giant component is larger in municipalities. (**d**) Number of links within the same tower using several randomization models. Results are averaged over 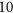 runs. The real network has a bigger number of intra-tower links than a space independent graph (ER) and a 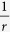 model. In the case of Lisbon, the real network has even more links than a 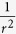 model. To explain the high number of intra-tower links the geographical distance is not sufficient, thus another effect like clustering is needed.

To better illustrate this finding we have studied all intra-tower networks in the capital cities and compared them to networks of the same size centered in municipalities in the countryside. Fig. 5a shows the average giant component for towers and municipalities of a certain size. Municipalities with a given population have a larger giant component than a tower in a city with the same population.

Given a fixed number of nodes, a giant component emerges more likely with a higher number of links and with low clustering (a link closing a triangle does not enlarge any connected component). As shown in Fig. 5b-5c, both effects are present at the municipality level and not within towers. This explains the different giant component sizes between municipalities and towers. However, high clustering seems to be dominant for the lack of a connected component, since in Portugal the average degree is the same in towers and municipalities. Moreover, the small average degree does not seem to be due to lack of data, since the data from France presents the highest average degree at a country scale, while it exhibits the smallest average degree on the tower scale.

Our results on geographic distance 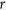 agree with previous literature^15^,^4^ showing that the probability of two users within distance 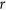 to be connected follows 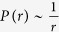. However, this sole finding does not give us any information about the number of links between people within the same location (tower/zipcode), since in principle they are within 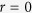 distance. In order to be able to apply pure geographical models (generating links with 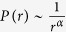) to our data, we have to randomize the position of the users around the tower’s location. A common assumption for mobile phone data is considering that if a call is processed by a tower, then that tower is the closest to the user’s location. This assumption implies that the geographic space can be divided according to the Voronoi diagram of the towers in that region. This way our randomization assigns each user a position uniformly distributed in the Voronoi cell it belongs to. Figure S22 shows the randomization process in Paris and Lisbon. After randomization, the distance 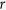 between any two users is greater than zero, so we can apply 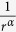 models the number of predicted and present intra-tower links for the same number of links in the whole network. In Fig. 5d we show that the number of observed intra-tower links in both cities is higher than what a pure geographical model 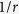 would generate (even higher than a 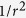 in the case of Lisbon). Despite this abundance of links, there is no giant component, what implies that clustering plays a major effect at this level, producing highly clustered *islands* within the same tower.

### Geographical dispersion of urban communities

**Figure 6:**
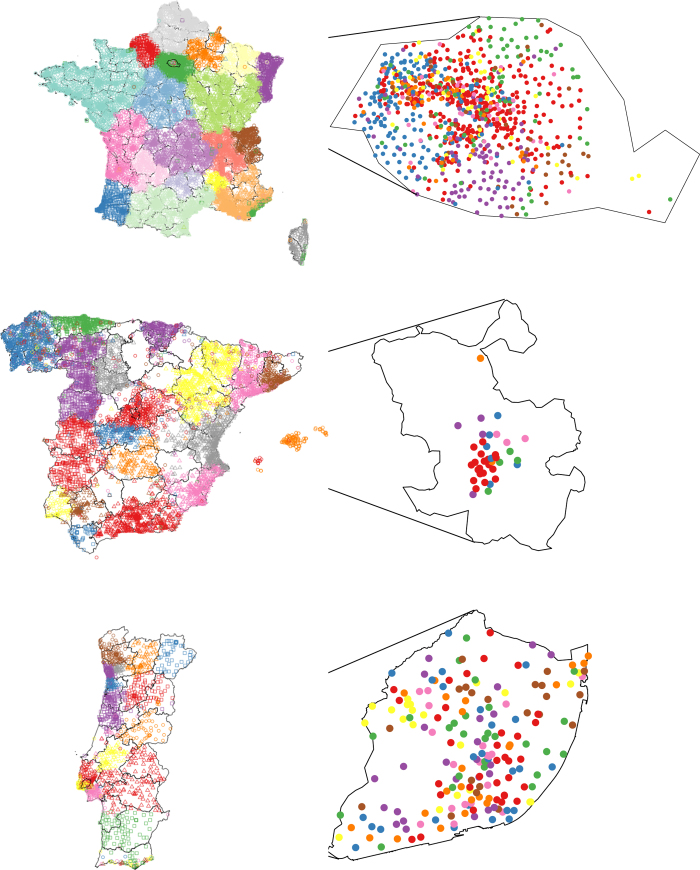
Geographical clustering of social communities. On the country scale, towers belonging to the 20 biggest communities are presented in different colors and shapes. On the city scale, towers within each capital city are presented. On the country scale most of communities fit with the administrative boundaries while within cities communities do not seem to be geographically driven. The figure was created using R packages *maptools* and *ggplot2*.

On the country scale the identified communities are known to be highly spatially correlated and even redraw the administrative borders as shown in Fig. 6 (left) where the colors indicate the dominant community of each mobile phone tower. This has been the motivation of a research line oriented to *redraw* the political maps according to social network features^18^,^20^,^46^. However, in the city scale (Fig. 6 right) the communities are dispersed over space and within the downtown area they are nearly randomly distributed. This shows for the first time that communities within cities are not geographically determined.

These results are confirmed by the measurement of 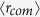 (average distance between two towers belonging to the same community) and 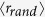 (average distance between two random towers), which are reported in table 2. Details on the calculation of both distances can be found in the **Methods** section. While 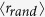 is consistently over 4 times larger than 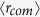 in the country scale, the two measures become much more similar within cities, quantitatively confirming the visual result on Fig. 6.

An additional unexpected finding is that some touristic areas break the general country-wide trend. A significant part of the French Riviera and the south coast of the island of Corsica belong to the Paris community, even if they are far away from the capital city. Same thing happens with Ibiza (western most Ballearic island) and Madrid. In Portugal’s Algarve (south coast of the country) the effect is not so clear, but there is definitely a higher community diversity in the area, and it is possible to find towers belonging to both Porto and Lisbon communities. Note that this is unlikely to be a touristic seasonal effect, because in France and Portugal the most used tower in a 6 month period is assigned to the user, and in Spain the billing zipcode is used. Since both are reasonable proxies for permanent residency, this effect is more likely due to urbanites who retired to the coast, and even become majority in certain areas, but still keep their social ties back in the large metropolis.

### Distance Metrics and Searchability in Urban Networks

Network searchablity is related to its links density^34^,^35^. The density of links 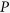 as a function of nodes distance 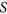 determines the necessary condition for network searchablity. This condition is postulated in the group model framework^35^, which generalizes previous results in hierarchies of social networks^34^ and spatial lattices^39^. 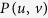 is the probability of link existence between a pair of nodes 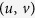 that are within distance 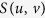, defined as the size of the smallest group containing both 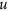 and 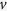.

Given the distance distribution of the form 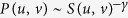 when 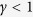 the social network is not searchable; if 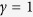 the social network is always searchable, and if 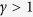 the network can be searchable.

**Figure 7:**
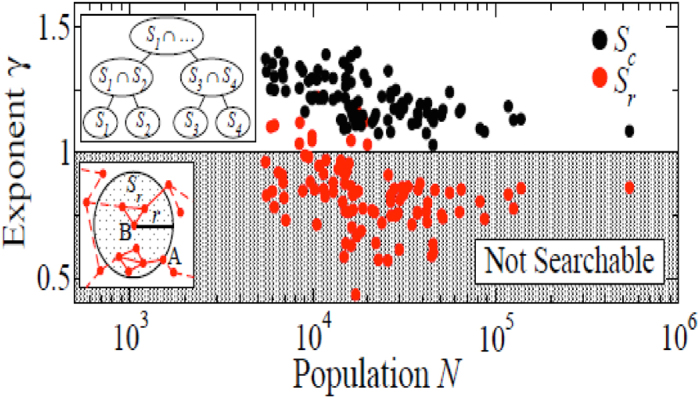
Comparison of the exponent 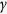 for the probability of finding a link between two people as a function of smallest common group size: 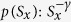 for 96 cities in France. Groups are constructed either based on geography (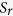, black) or on community (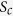, red).

We test this structure in our data using geometric and social distances. We divide the network into groups of size 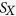 using either geographic balls of a certain radius 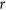 (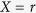) or existing communities (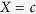) as illustrated in the insets of Fig. 7. Then we calculate the probability that two nodes that belong to the same group (being that group the smallest they both belong to) share a link and how this probability depends on the group size. We observe that both functions have the exponent close to 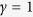, but in the groups based on geography these exponents are always below 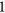, while the exponent is consistently above 1 for communities as shown in Fig. 7. Although the group-model framework does not capture all of our network properties (heterogeneous degree distribution and clustering coefficient) we find that our empirical results in urban networks confirm theoretical results regarding the conditions for searchability of social networks.

## Discussion

In summary, we have demonstrated that cities (as defined conventionally by their administrative borders and population size) change the structure of social networks. Interestingly, these findings could be related to urban growth and the economic function of cities^23^,^24^.

Taken together, the presented results lead to the following discoveries: (i) Communities within cities follow a hierarchical structure that favors social distance over geographic distance. (ii) While people living within geographic radius including several cities form a connected network, the same radius within cities leads to highly clustered components only connected through people in distant parts of the city. This behavior occurs across different cities and regions sizes, highlighting cities as functional entities of the social networks (iii) The structure of communities (here related to social proximity) and not geographic distance is what makes social networks searchable within cities. This finding is consistent with experimental results that suggest people do use the profession or name of the target in the final steps to make inferences about his/her education or ethnicity, as a hint to help routing within cities^33^.

This work uncovers an unknown feature of social networks: while at the national level descriptions of social networks consist of highly connected and geographically close communities, we find that geography plays only a minor role when forming communities within cities. Urban networks consist of geographically dispersed communities. This structure explains why people are able to successfully route in Milgram-like experiments, provided they correctly identify the community of the target. Our results support the theoretical hypothesis of Kleinberg: the likelihood to find friendships within communities decays as a power-law with increasing community size^35^, confirming that among all possible network configurations, humans have favored those such that a message can reach anyone even if delivered using only local information. This is a remarkable example of a self-organized structure that allows a small group of individuals to solve a complex problem by cooperating to take advantage of collective knowledge^47^,^48^.

## Methods

### Data

We analyze phone records for a six months period in three countries: France, Portugal, and Spain. In total 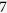 billion phone interactions are considered. In order to build social networks from this data, only links with at least one communication per direction are included. This is a common technique in the literature^7^,^42^,^49^ to avoid both marketing callers and misled numbers. The resulting social networks have 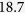, 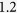, and 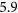 million users, for France, Portugal, and Spain respectively. Further details are provided in the SI.

### Routing Algorithms

In order to deliver the message, several strategies can be used. In the following we describe every criteria used in our experiments.

#### RAN

We use random routing as a baseline comparison, by employing depth first search (DFS) into a routing algorithm, we effectively avoid the message to get into infinite loops. The application of DFS in the Milgram experiment is quite straightforward: when a participant receives a message, he/she knows the list of people who already got the message. The participant will never forward to none of these people, unless all of his/her friends are in the list. In this case, he/she will send the message back to the person who first sent the message to him. In a tree network, this would be the case of a branch which has been explored without success and the search process continues going backwards. Since our social network is far from being a tree, the number of rolling back events is low (less than 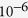 in all of our simulations).

#### GEO

This procedure consists of sending the message to the friend geographically closest to the target. In the intercity scenario, locations are considered on the municipality level. In the intracity scenario, tower locations are employed. Note that this discretization produces a number of ties (two or more friends are at the same distance from the target).

#### DEG

In this case, the message is forwarded to the friend with the largest number of friends among the candidates.

#### COM

In order to mimic social attributes (school, work) communities are detected in the network. To detect communities in social networks, we use the well-established Louvain method^17^,^37^,^49^,^50^,^51^. This method is a greedy optimization method that attempts to optimize the network modularity by aggregating nodes belonging to the same community and building a new network whose nodes are the communities. This method assigns to each person a set of communities at different hierarchical levels. Although the number of aggregation levels 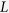 depends on the network and it is automatically obtained from the algorithm, in all of our networks the algorithm provided between 3 and 7 aggregation levels. Note that this algorithm provides hierarchical communities. If two nodes 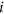 and 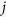 have a community of level 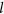 in common they will share as well all the communities in higher levels, formally:


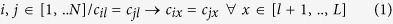


where 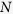 is the number of people. A person will send the message to a friend with the lowest possible community level in common with the target. While it is arguable that community detection requires global information and such might not be available to participants in a Milgram-like experiment, recent research^52^ has reported that people are able to relate communities detected in their network to certain social attributes and affiliations, thus making communities a reasonable proxy for those unknown attributes in our data set.

In our experiments, these criteria are combined, by using several of them to solve ties: this way, we will denote *ran-deg* to a routing scheme where first the already visited nodes are discarded from candidates (*ran*), and then those with the highest degree are chosen (*deg*). If there is still more than one possible friend after the routing logic is completed, the message is forwarded to one of these candidates at random. In our *ran-deg* example, this happens if two or more friends were not previously visited and have the same degree.

### Geographical dispersion of communities

We found a fundamental difference between the behavior on urban scale and on the country one: geographical clustering turns out to be more intense in the intercity scenario than in the intracity one. To reach this conclusion we have calculated the spatial clustering of the communities by the following steps:

- Perform a community detection on the network
- Associate the tower to the most common community among that tower’s users.
- Calculate the average distance 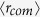 between any two towers belonging to the same community
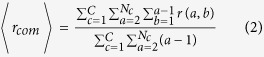
where 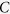 denotes the number of communities found, 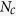 the number of towers in community 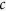 and 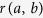 the distance between towers 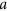 and 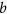.
- Assign communities with the same sizes randomly to the towers and calculate the average distance (2) of the randomized data 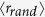.

## Additional Information

**How to cite this article**: Herrera-Yagüe, C. *et al.* The anatomy of urban social networks and its implications in the searchability problem. *Sci. Rep.* **5**, 10265; doi: 10.1038/srep10265 (2015).

## Supplementary Material

Supplementary Information

## Figures and Tables

**Table 1:** Characteristic properties of the social networks in the studied countries: Size of the giant component (GC), number of users (Nodes) and relationships (Links), average degree 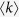 , average clustering coefficient 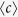 , average shortest path length 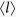 , and the corresponding values for random networks with the same size 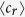 and 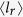 .

Country	**% GC**	**Nodes** 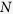	**Links** 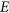	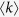	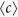	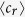	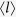	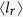
France	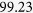	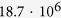	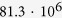	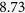	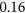	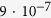	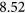	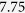
Portugal	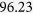	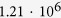	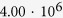	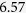	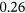	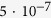	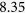	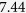
Spain	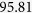	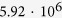	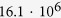	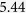	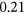	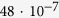	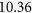	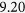

**Table 2:** Average distance between two towers belonging to the same community ( 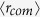 ) compared to the distance when the communities are randomized ( 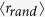 ). The geographical effect 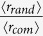 is more pronounced in the nation-wide communities.

Network	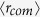 **(km)**	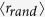 **(km)**	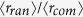
Portugal	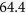	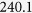	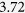
France	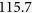	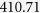	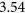
Spain	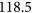	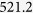	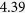
Lisbon (*concelho*)	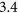	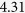	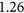
Paris (*department*)	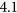	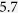	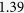
Madrid (*municipio*)	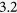	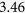	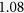
