# Supplementary material for: The anatomy of urban social networks and its implications in the searchability problem
Source: Sci Rep. 2015 Jun 2;5:10265. doi: 10.1038/srep10265 (PMC4451794; doi:10.1038/srep10265)
Supplement: Supplementary Information [file srep10265-s1.pdf]

Supplementary Information: The anatomy of urban social networks and its implications in the searchability problem

C Herrera-Yagüe CM Schneider T Couronné
Z Smoreda RM Benito PJ Zufiria MC González

1 Data description

Our data contains phone records for a six months period in three countries: France, Portugal, and Spain. In total 7 billion interactions are considered. In order to build the social network, only links with at least one communication per direction are included. This is a common technique in the literature [30, 29, 21] to avoid both marketing callers and misdialed numbers. After applying this filter, the network presents the characteristics shown in table 1 in the manuscript.

	α	k_{min}	KS.stat	KS.p
Portugal	5.19	73.00	0.010	0.97
Spain	6.22	32.00	0.005	0.93
France	4.88	33.00	0.012	0.00

Tab. S1: Results of truncated power law fits for the degree distribution of all 3 networks. For Spain and Portugal, a power-law can be fitted in the tail of the distribution. The fit has been done using the procedure described at [10].

1.1 User location

A key aspect in the creation of a link between two individuals is the geographical distance between them. In our study, users are located in their billing zip code (Spain) or their most used tower (France and Portugal). In total 8928 different locations are available in Spain[1], 17475 in France and 2209 in Portugal. Figure S1 shows the distance distribution to the first, second and third closest zip code or tower in the three datasets. Although towers may provide a slightly more accurate geolocation, both are sufficient for our purposes.

[1] Spain zip codes are geolocated according to geonames database, available at http://downloads.geonames.org/export/zip, and grouped according to latitude and longitude since some zip codes have identical coordinates. Towers coordinates were provided by the carrier.

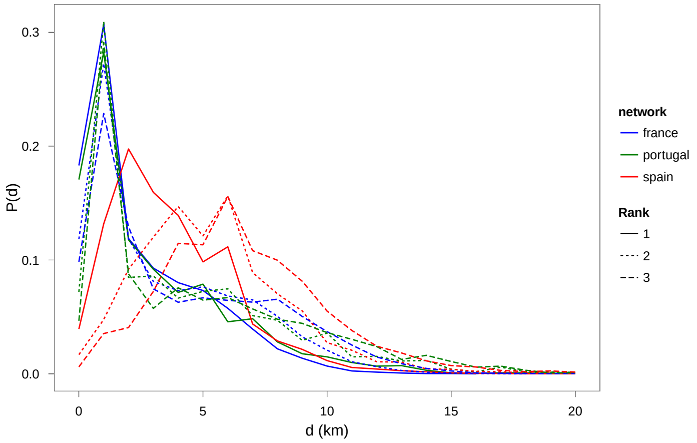

Fig. S1: Distance distribution to the first, second and third closest tower or zip codes. Towers (France and Portugal) are slightly closer to each other than zip codes (Spain) are.

On the other hand, users are not equally distributed among towers and zip codes. Figure S2 shows the cumulative distribution in the three data sets. Most of the towers serve between 100 and a few thousands users, while zip codes' user count is more heterogeneous (the maximum is a zip code in Madrid with 125,000 users). The explanation for these different results comes from technical reasons: as the demand rises in an area, additional phone towers need to be installed to handle the traffic.

For simplicity, from now on we will refer both towers and zip codes as towers, unless otherwise mentioned to explain different results among different data sets.

1.2 Sampling effects

Users in the network are not homogeneously distributed, in some regions there is a slightly higher concentration. This variance may come from a higher market share of the mobile phone provider or from a higher usage of mobile phone service in the area (only users who have at least one mutual relationship appear in the network). The differences between different regions are depicted in figure S3.

We refer user density as the ratio $u_i = \frac{\text{Users}}{\text{Total population}}$ in a certain region i. The main effect of having different u_i seems to be in the average degree of the resulting subnetwork. Figure S4 shows this relationship, which turns out to be close to linear. For a network where all inhabitans are present (i.e, $u_i = 1$), a projection of the resulting linear model would be $\langle k \rangle \simeq 16$.

In any case, the number of contacts in a phone network is relatively small compared to other social networks obtained from online social sites (average degree are in the hundreds [25, 15]) or compared to different figures proposed

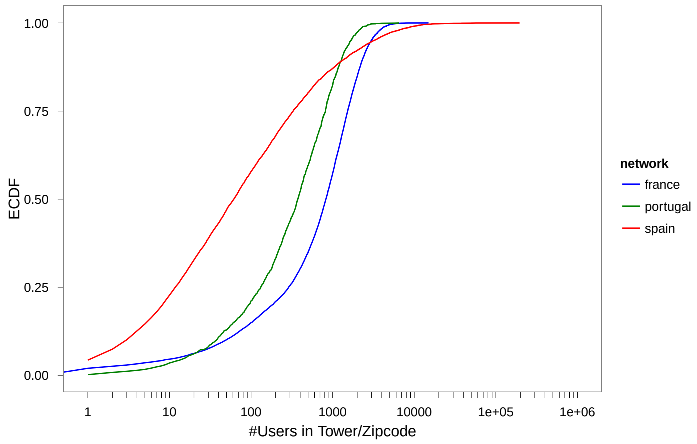

Fig. S2: Empirical cumulative distribution function of number of users in each tower or zip code. Due to technical reasons, the range of users per location is smaller when towers are used, while zip codes distribution is more broad.

as average degree for humans: extrapolation from observed correlation between social group size and neocortex volume in primates drove Dunbar to propose 150 [12], while recent statistical estimation methods based on self-reported data range between 290 [23] and 610 [24]. We will show that increasing the average degree has a positive effect on routing, which means any result we get by studying the phone social network can be considered as a lower bound for the real world's social network. On the other hand, the phone network can be seen as the backbone of the social network, since it contains only interactions the people are willing to pay for.

2 Intercity routing experiment

2.1 Assignation of user to cities

Our first experiment consists of, given a random pair of nodes in the network A and B, trying to deliver a message from A to the area where B lives. For systematically delimiting this "area where B lives" we have chosen administritative division over a regular spatial grid, because the resulting modularity in the social networks is significantly higher. Specifically, we will study two levels of agreggation in each of the networks:

- We will generically refer as provinces to the following administrative divisions: *départements* in France, *provincias* in Spain and *distritos* in Portugal. This way we divide the country into 97, 50, and 20 provinces, respectively. According to official census, the population ranges from 77

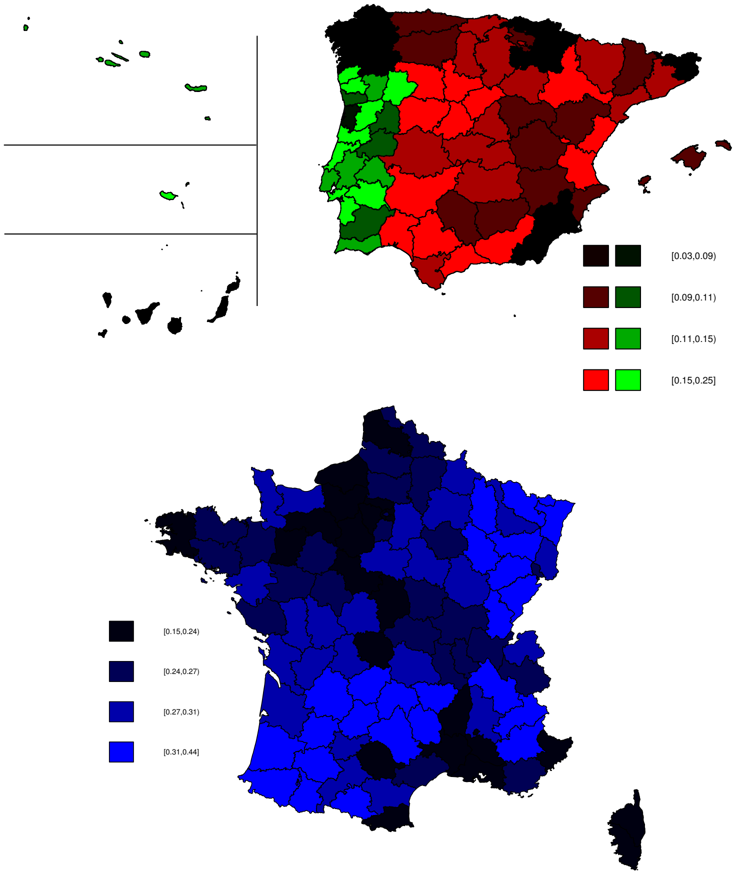

Fig. S3: Users/Population ratio in the province level. Brighter colors represent a higher ratio. The maps were created using the package *maptools* for R.

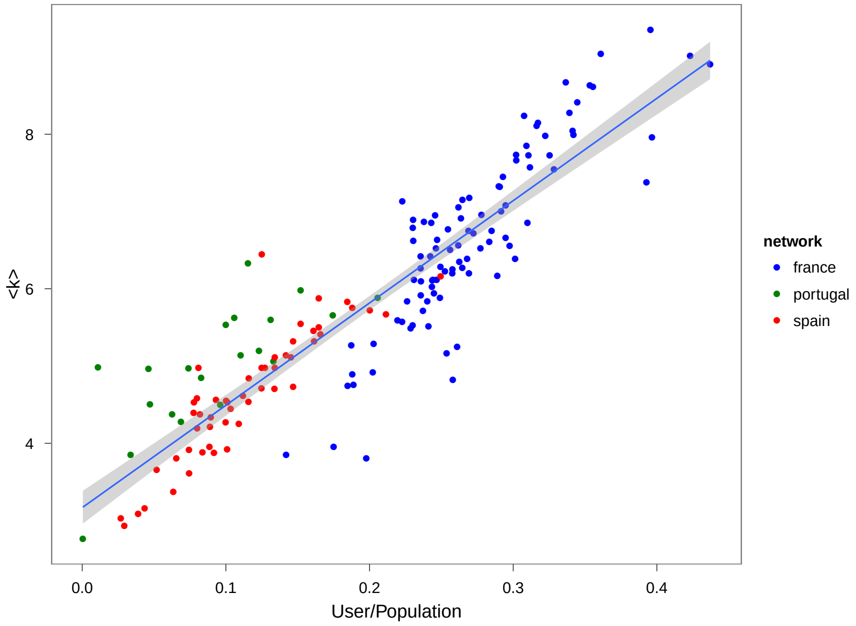

Fig. S4: Dependence of the average degree $\langle k \rangle$ on the ratio between users and population. Each point represents a province network. It can be appreciated how closely related $\langle k \rangle$ and $u_i = \frac{\text{Users}}{\text{Population}}$ are. Blue line presents a linear fit $\langle k \rangle = 3.16 + 13.24 u_i$ where $R^2 = 0.818$.

a) b)

Fig. S5: Provinces (a) and municipalities (b) map of the three studied countries. The maps were created using the package *maptools* for R.

thousand (Lozère, France) to 6.4 million (Madrid, Spain). A province map for all three countries is depicted in figure S5a.

- We will generically refer as municipalities to the following adminsitrative divisions: *cantons* in France[2], *municipios* in Spain and *concelhos* in Portugal. Our users are located in 3520, 5446 and 297 different municipalities respectively. A map depicting municipalities in all three countries is presented in figure S5b.

To map the user coordinates into the appropiate divisions we have used Global Administrative Divisions database[3] except for France's *cantons,* where the GEOFLA database by IGN has been used[4].

2.2 Experiment conditions

Once we have assigned users to their cities, we ran the experiment in the following setup: in each country we chose 60 thousand random source and targets among all nodes in the network. Next, we try to deliver the message using combinations of techniques described in section Methods in the manuscript. Additional to those, we have performed a pure *geogreedy* (passing to the geographically closed friend, and if no one is closer than the current user, consider the chain broken) as well as the modification proposed in [22], which we have

[2] We have used this division instance of the *communes* because of the high number and high heterogenity of the latter (over 36 thousand diffents *communes,* ranging from 10 people to 2 million). Most of *cantons* are composed of several *communes*, being Paris a special case: Paris city actually fills the whole departement 75, and is divided into 20 *arrodisements* (districts) which are counted as cantons. In any case, when we refer the Paris city in the intracity network experiment, we mean department 75. Some other large french cities are also divided into several cantons.

[3] http://www.gadm.org/

[4] http://professionnels.ign.fr/geofla

denoted *geogreedy++*, and consist of forwarding the message to another user in the same location even if she is not connected to the current user. For each pair and algorithm, up to 1000 hops are simulated before reaching target's city.

2.3 Experiment results

First of all, in intercity routing, using provinces as target seems to make the routing process trivial (even random routing delivers a significant amount of messages), so we will present the results of the routing trying to reach the right municipality. The main conclusion is that any routing strategy other than random will deliver the messages with a high probability (as we can see in figure 2a in the main text). If we study small differences in error rate after 100 steps between the algorithms (see figure S6) we find statistically significant differences between the algorithms. In general, *geo* methods outperform *com* methods, and solving geographical ties (two people are at the same distance from the target) using degree increases routing performance. Another relevant finding is that these distributed routings reflects the same behavior than the optimal routing: it is harder to route in Spain (due to the smallest average degree) than in France, despite the number of nodes in France is about 4 times larger.

In order to provide a more detailed look of this experiment, we have published the following webapp: www.someurl.com. In the app, the user can pick among 180 thousand routes we have simulated, choosing first target city and then source. To illustrate the difference between distributed an optimal routing, both optimal and best decentralized (*ran-geo-deg*) routes are plotted, and also the number of nodes explored to find the optimal path is presented. Theoretically, a ran can go "backwards" in the exploration of the network if all friends have been already visited, producing a loop in the sequence of explored nodes. However, we did not find evidence for this in our simulations (overall, over 3.2 million hops were simulated). In figure S7 an snapshot of the app is presented, with one route as an example. On average, in France, the distributed routes found have 18.1 hops, while 7.2 hops are optimal. However, in order to find the optimal routes on average 8.1 million nodes have to be explored.

Besides, we have studied how the size of target's city influences the length of the distributed route found. Intuitively it is easier to reach a big town like Madrid (3.2 million inhabitants in the municipality and half million users in our network) than a small city with just a few hundred inhabitants. However, our results show how the size of the destination city affects only logarithmically to the length of the route found to reach them (see figure S8).

3 Intracity experiment

For the intracity experiment, we have divided the country networks into both provinces and municipalities networks. All provinces have been studied, and the

[6] OpenStreetMap Copyright and License http://www.openstreetmap.org/copyright (Date of access:28/11/2014).

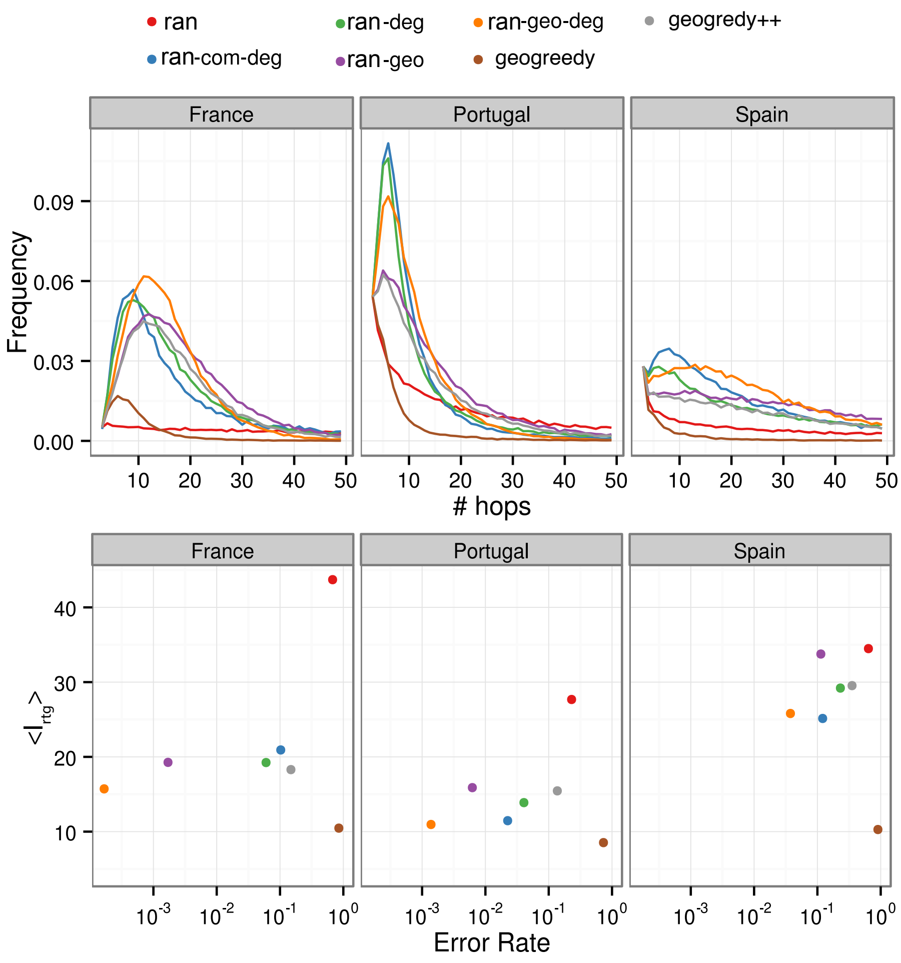

Fig. S6: Intercity performance for different routing algorithms. Top graph shows the fraction of messages arriving at the target in the first 50 hops $f(n)$. Since the fraction of messages decreases with the number of hops, one could evaluate the performance by measuring the mean and the integral of this distribution after N hops, with N being large enough. The bottom graph presents the average path length of the delivered messages $\langle l_{rtg} \rangle = \sum_{n=1}^{N} n f(n)$ and the fraction of failed messages $E = 1 - \sum_{n=1}^{N} f(n)$ for $N = 100$.

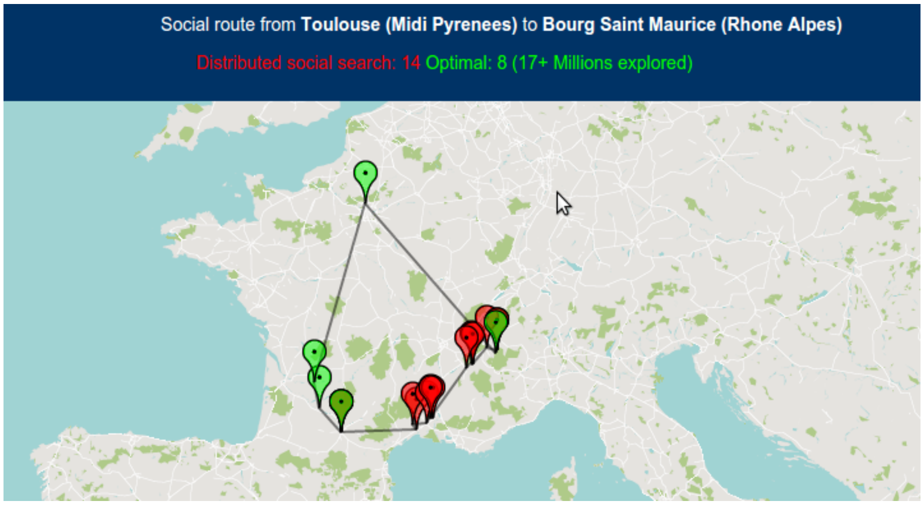

Fig. S7: Snapshot of the app we developed for visualize our results. The red route is the result of distributed *ran-com-deg* while the green one displays the optimal route. In this example, the distributed route needs 14 steps to reach the destination city, while the optimal route uses 8. However, the distributed algorithm explores only 123 nodes, while more than 17 million nodes are checked for finding an optimal route. Figure created using using map tiles from openstreetmap.org (OpenStreetMap contributors[6], licensed under Creative Commons BY-SA 2.0 licence. To view a copy of this license, visit creativecommons.org/licenses/by-sa/2.0).

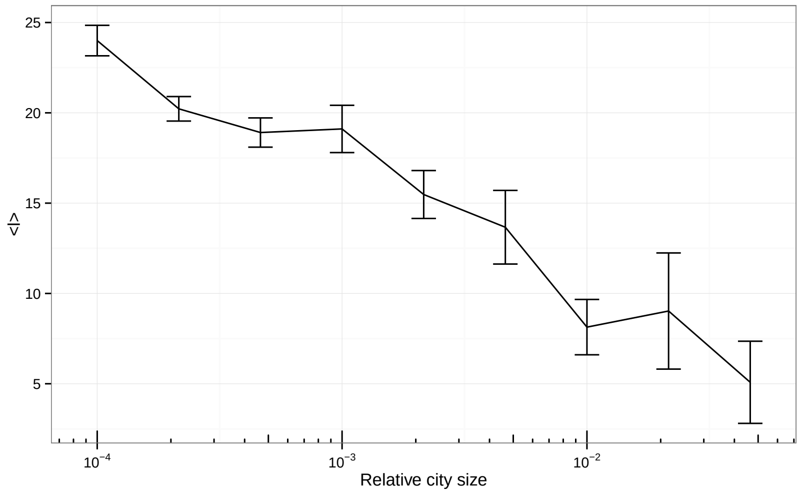

Fig. S8: Average number of hops needed to reach each city versus city size (relative to the size of the country's network). Error bars show the standard error of the mean.

100 most populated municipalities in each country (300 municipalities and 168 provinces in total). Province networks are almost connected (over 95% nodes in the giant component) and municipalities have also a quite big giant component (over 80%). In any case, these administrative boundaries produce significantly larger connected networks than any regular spatial grid. The reason for this is that the classification of nodes in either provinces or municipalities is indeed a good community classification (modularity[7] scores over 0.4 and 0.5 respectively) probably due to the high clustering of our networks. For the routing experiment, we take into account the nodes in the giant components (a path between any given two nodes actually exists) just as we did with the country networks.

We repeat the experiment in each of the networks with the same setup we used for the intercity experiment. In this case 100 thousand random pairs are simulated for the algorithms presented in figure 2c in the main text, while for all other algorithms, 10 thousand pairs are considered.

3.1 Results analysis

In figure S9 we present the routing results for the three capital cities (in fact these are worst case scenarios, since the networks are the largest). Figure S9 presents both $P(l_{rtg})$ distributions and their equivalence in the $(\langle l_{rtg}^{100} \rangle, E^{100})$

[7] Modularity is a standard metric to evaluate performance of community detection method, defined in [27] as $Q = \frac{1}{2m}(\sum_{ij} A_{ij} - \frac{k_i k_j}{2m})\delta(i,j)$ where A is the adjacency matrix of the network, k_i is the degree of vertex i and $\delta(i,j) = 1$ if i and j belong to the same community and $\delta(i,j) = 0$ otherwise.

plane, which we will use for comparison. In figures S10-S15 we include results for the top 20 provinces and municipalities in each country. Careful observation of these graphics allows us to draw the following conclusions:

- Algorithm ranking from best to worst, is almost constant over all studied networks.
- Among *ran* methods (algorithms avoiding loops), $\langle l_{rtg} \rangle$ and E are fairly correlated. If an algorithm A outperforms another algorithm B by finding smaller $\langle l_{rtg} \rangle$ it will also provide a smaller error rate. Thus, we can compare algorithms by using only one of the two metrics. In figure S16 we show the relation between these two metrics for the *ran-com-deg* algorithm.
- Contrary to what takes place in the intercity scale, using geography to route within the city does not produce efficient routing. Consistently over the network sets we study, community based routing *ran-com-deg* significantly outperforms *ran-geo-deg.* Interestingly, having additional geography information besides the community structure (this means there is more information to make the routing decision) seems to be misleading, specially in large networks, as it can be observed in the performance of the *ran-com-geo-deg* routing strategy.
- Among all algorithms tested, *ran-com-deg* is the one producing the best results.

3.2 Efficient routing and average degree

Networks are considered to be *small-worlds* if they have a high clustering coefficient (ratio between closed triangles and connected triples), and at the same time the shortest path length scales with the number of nodes in the network N like $O(\log N)$ [36]. A routing algorithm is considered to be efficient if it is *polylogarithmic* [19]: i.e, it is able to find, between any two nodes, a path of length $O(\log^{\alpha} N)$ with a high probabilty.

Then, we check if our *ran-com-deg* is in fact an efficient routing algorithm. In figure S17(top) we show the relation between network size and error rate. Although in most networks we find that the error rates depends logarithmically on the number of nodes, we see a number of outliers. We find these outliers have small average degree. In fact, the majority of networks that do not lie in the $O(\log N)$ behavior have average degree smaller than 4. Although to the best of our knowledge there is no previous result in the literature to explain this finding, we suggest the following explanation. In a random graph where all nodes have the same degree k, $k \geq 3$ is needed to be able to find paths $O(\log N)$[7]. On the other hand, recent work in the effect of clustering in percolation studies show how a growing transitivity implies a higher average degree is needed for the emergence of a giant component[28, 1, 4]. Since having a connected nework is a necesary condition to route, we conclude our empirical observation is consistent

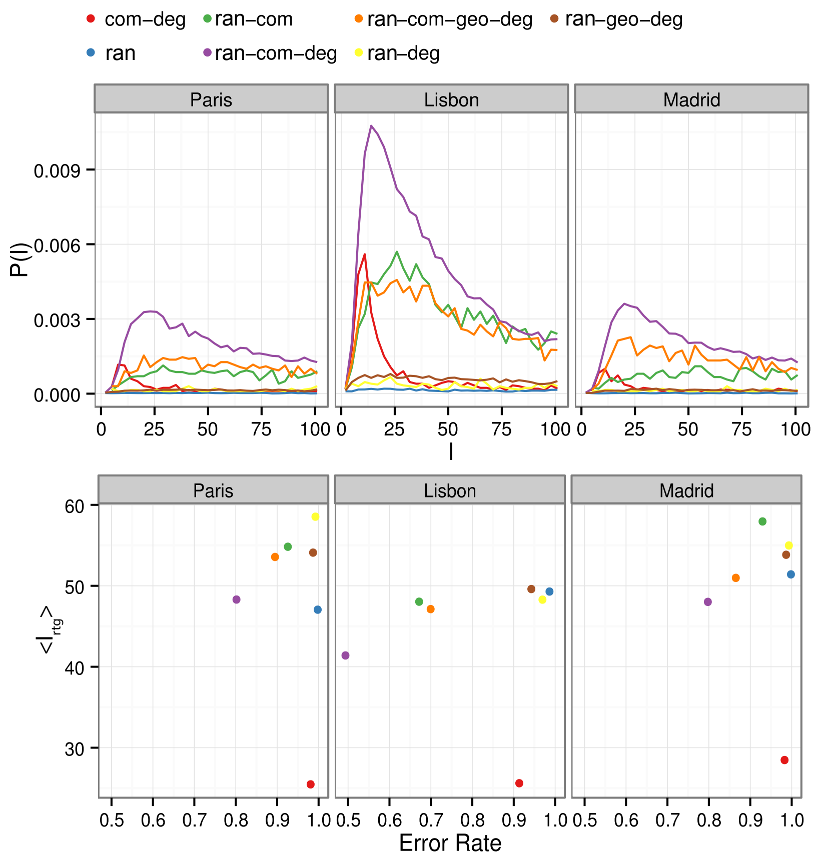

Fig. S9: Intracity experiment results for the 3 main cities. Top graph shows the fraction of messages arriving at the target in the first 50 hops $f(n)$. Since the fraction of messages decreases with the number of hops, one could evaluate the performance by measuring the mean and the integral of this distribution after N hops, with N being large enough. The bottom graph presents the average path length of the delivered messages $\langle l_{rtg} \rangle = \sum_{n=1}^{N} nf(n)$ and the fraction of failed messages $E = 1 - \sum_{n=1}^{N} f(n)$ for $N = 100$.

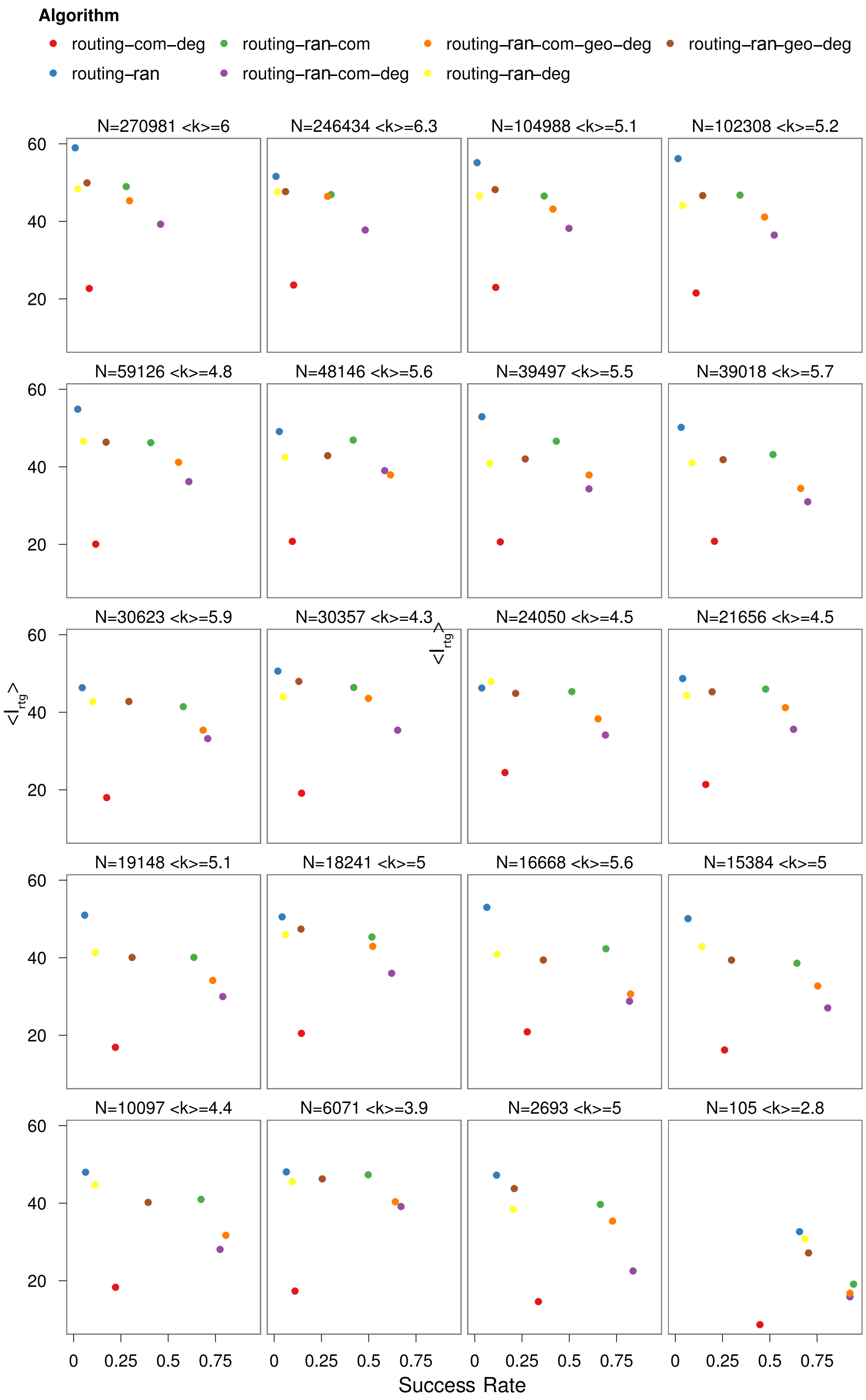

Fig. S10: Intracity results for the 20 biggest provinces in Portugal. N denotes the number of nodes, and $\langle k \rangle$ the average degree. Success rates refer to the proportion of messages delivered after 100 steps and $\langle l_{rtg} \rangle$ to the average path length of successful chains.

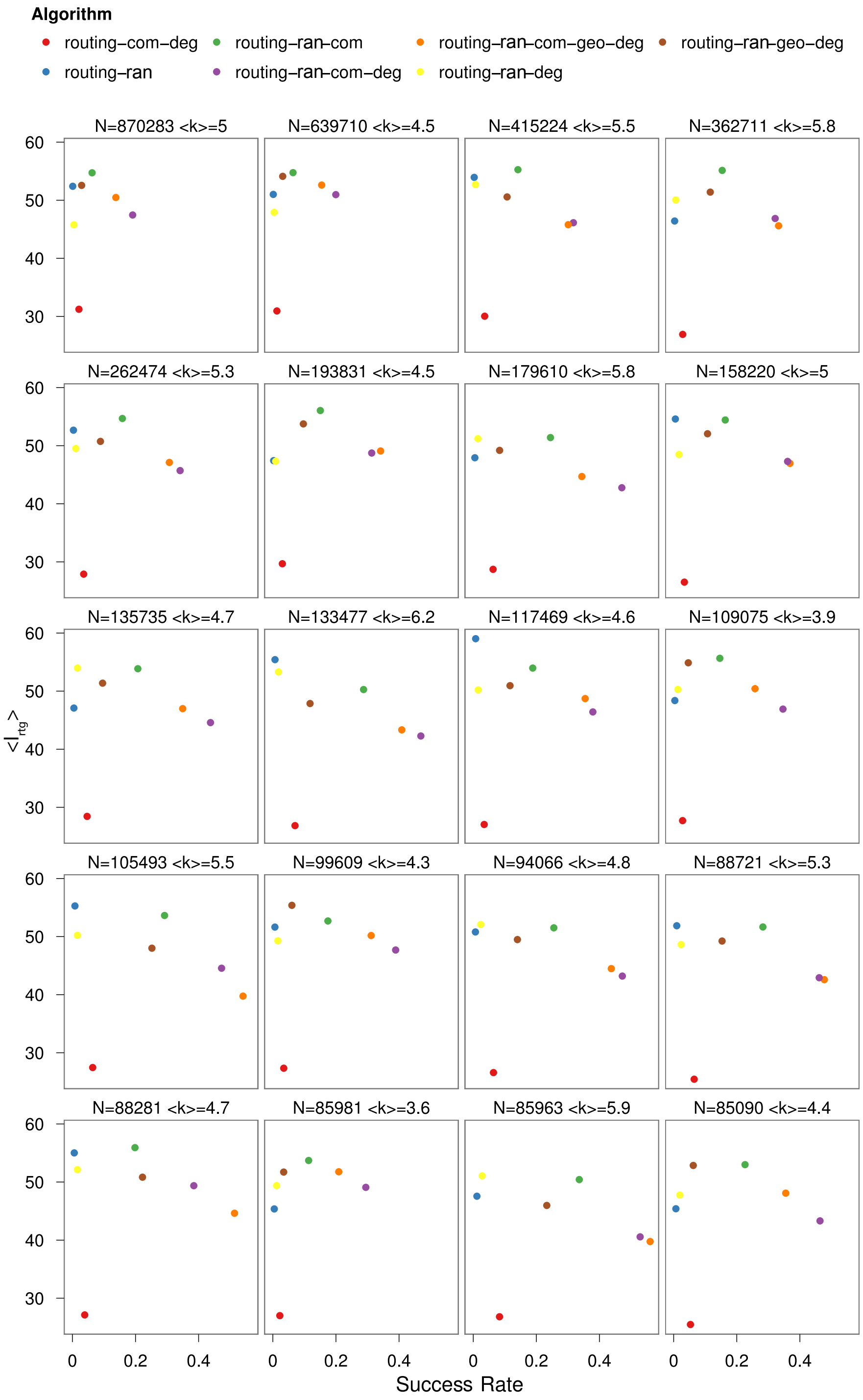

Fig. S11: Intracity results for the 20 biggest provinces in Spain. N denotes the number of nodes, and $\langle k \rangle$ the average degree. Success rates refer to the proportion of messages delivered after 100 steps and $\langle l_{rtg} \rangle$ to the average path length of successful chains.

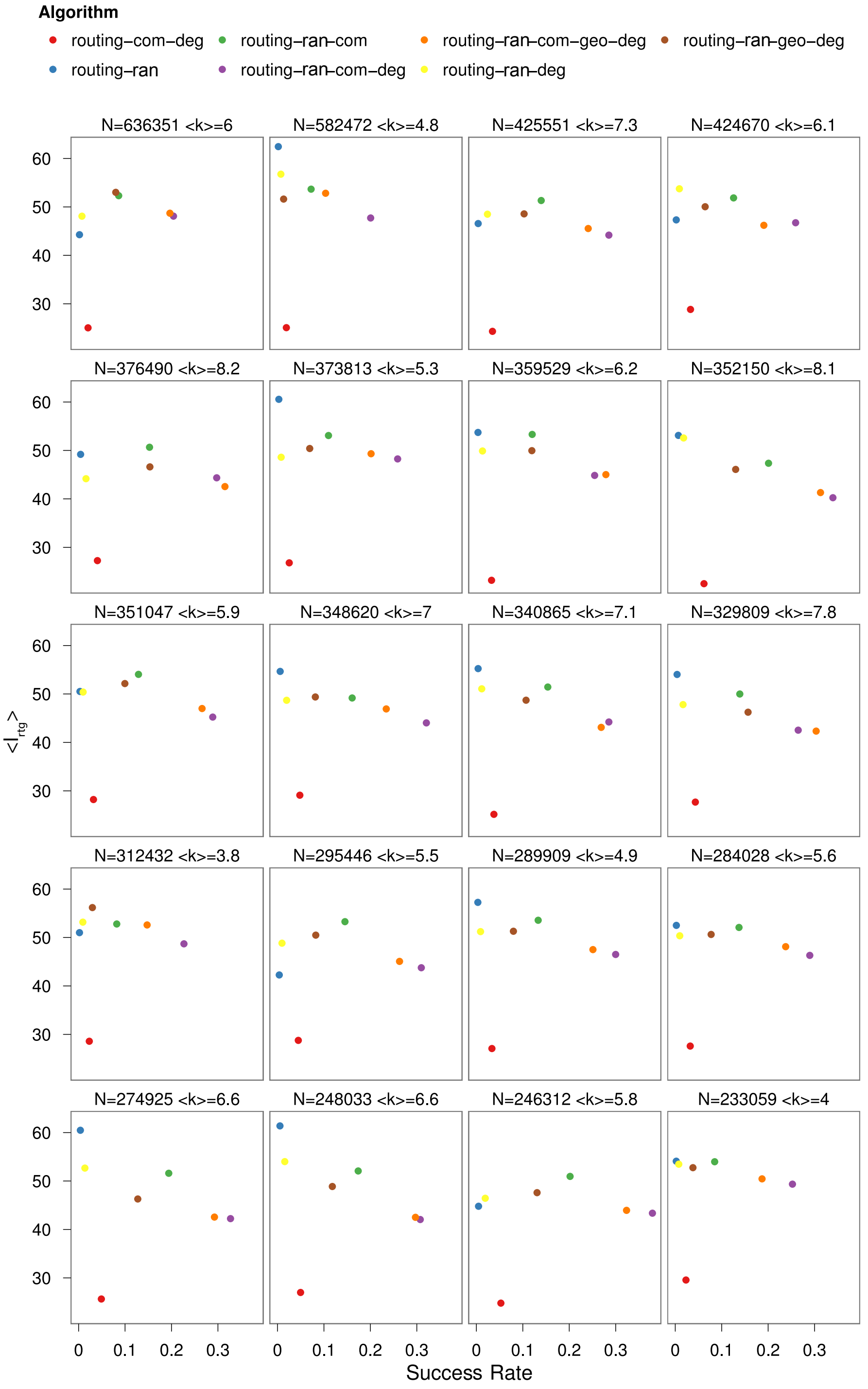

Fig. S12: Intracity results for the 20 biggest provinces in France. N denotes the number of nodes, and $\langle k \rangle$ the average degree. Success rates refer to the proportion of messages delivered after 100 steps and $\langle l_{rtg} \rangle$ to the average path length of successful chains.

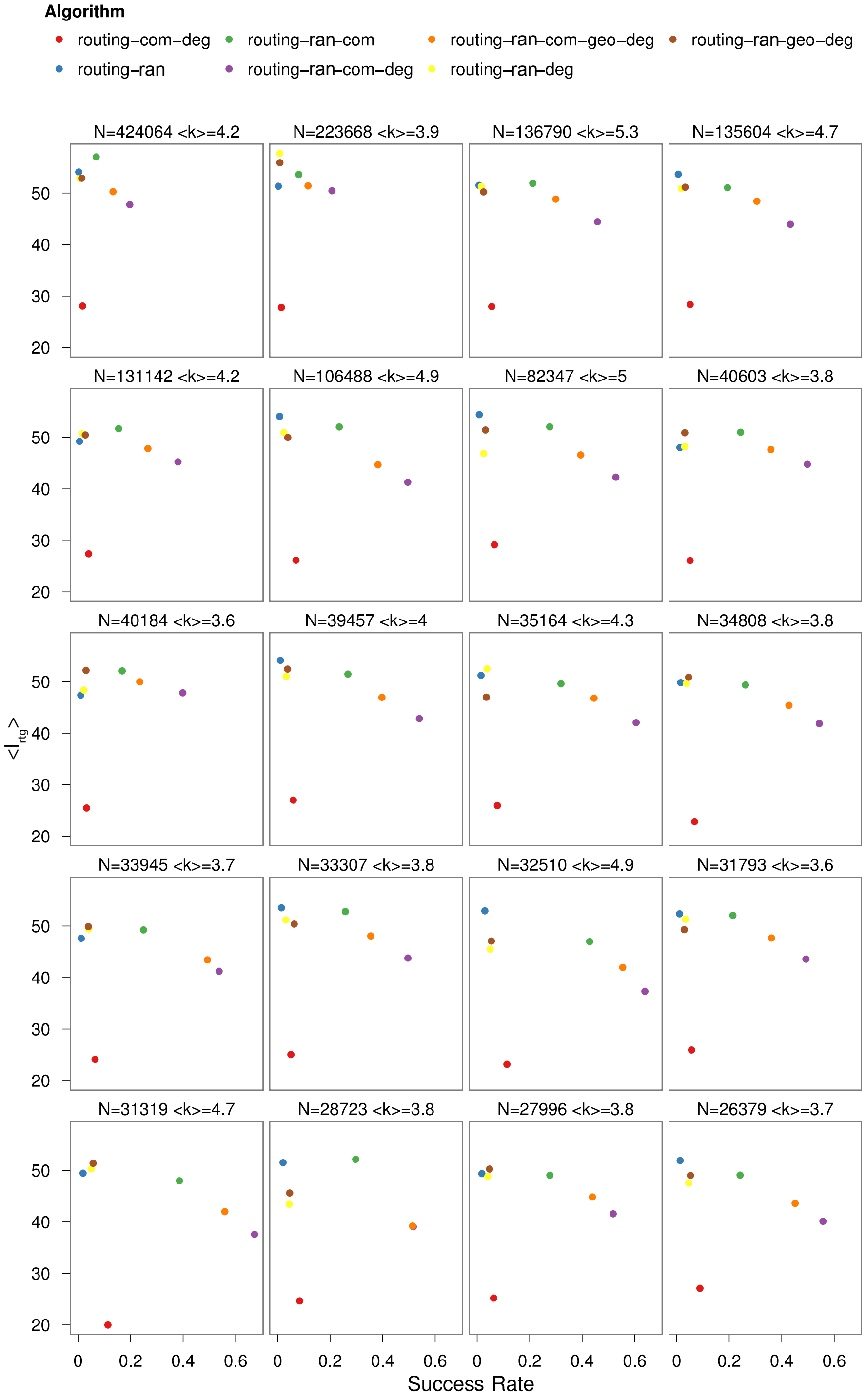

Fig. S13: Intracity results for the 20 biggest municipalities in Portugal. N denotes the number of nodes, and $\langle k \rangle$ the average degree. Success rates refer to the proportion of messages delivered after 100 steps and $\langle l_{rtg} \rangle$ to the average path length of successful chains.

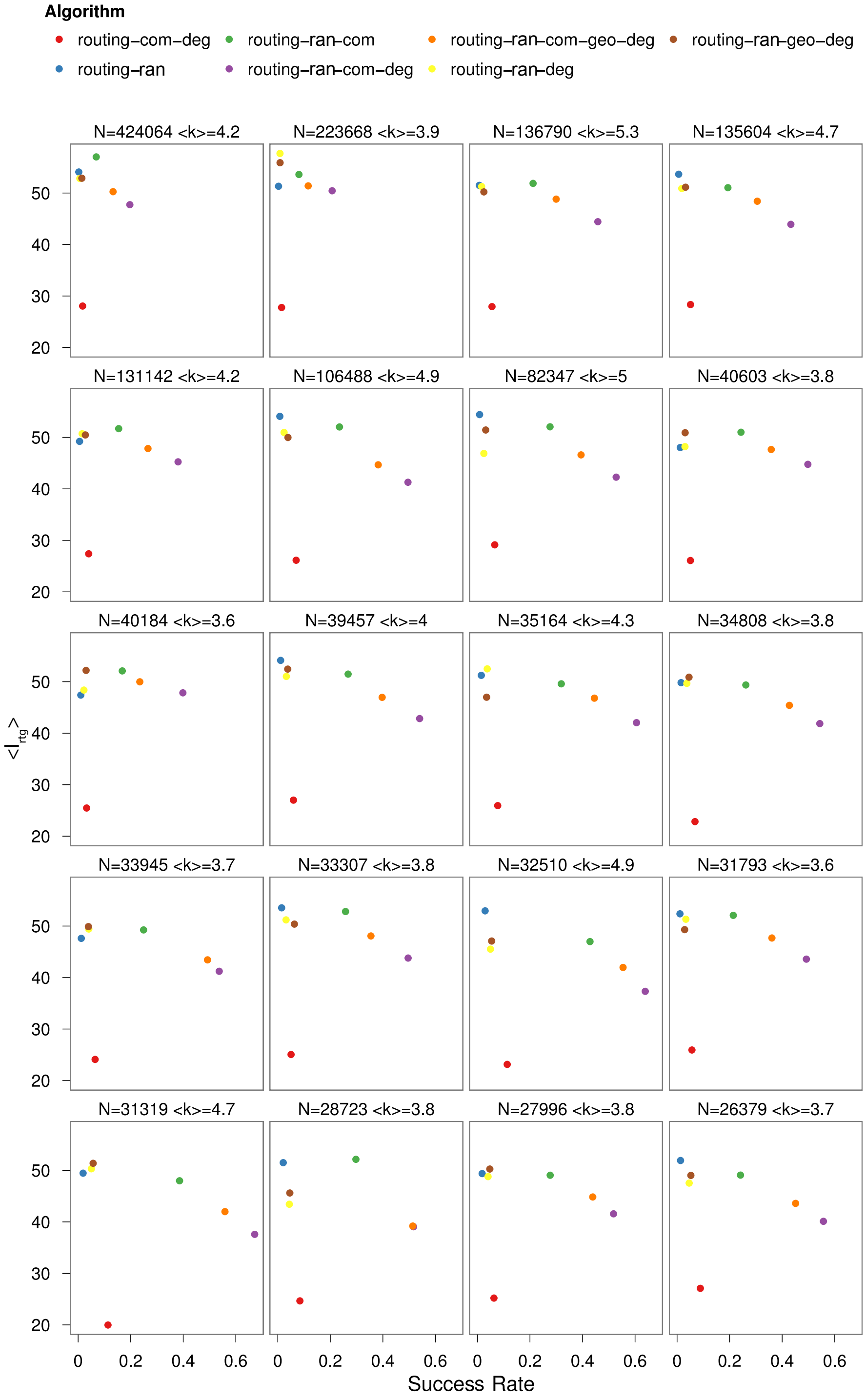

Fig. S14: Intracity results for the 20 biggest municipalities in Spain. N denotes the number of nodes, and $\langle k \rangle$ the average degree. Success rates refer to the proportion of messages delivered after 100 steps and $\langle l_{rtg} \rangle$ to the average path length of successful chains.

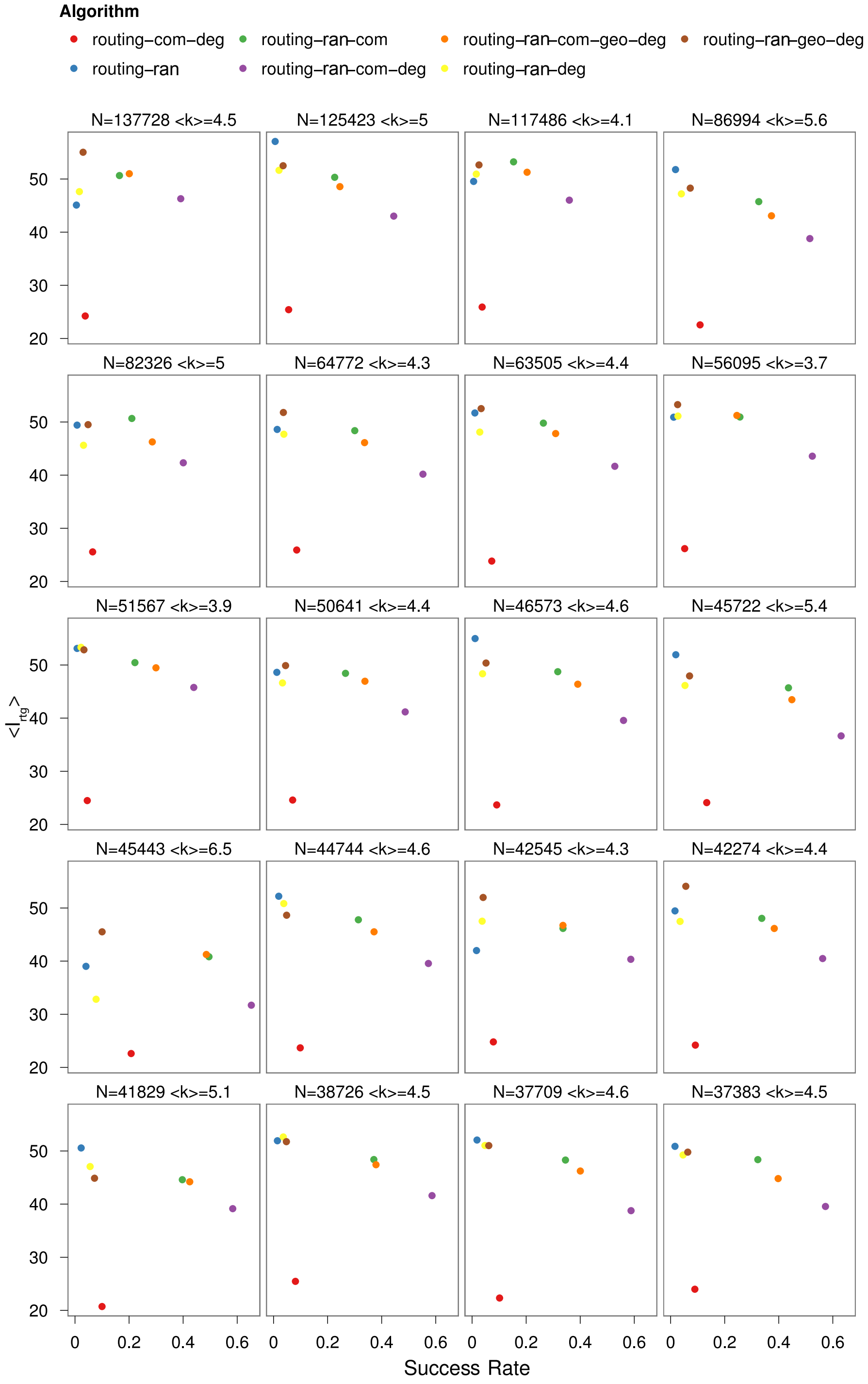

Fig. S15: Intracity results for the 20 biggest municipalities in France. N denotes the number of nodes, and $\langle k \rangle$ the average degree. Success rates refer to the proportion of messages delivered after 100 steps and $\langle l_{rtg} \rangle$ to the average path length of successful chains.

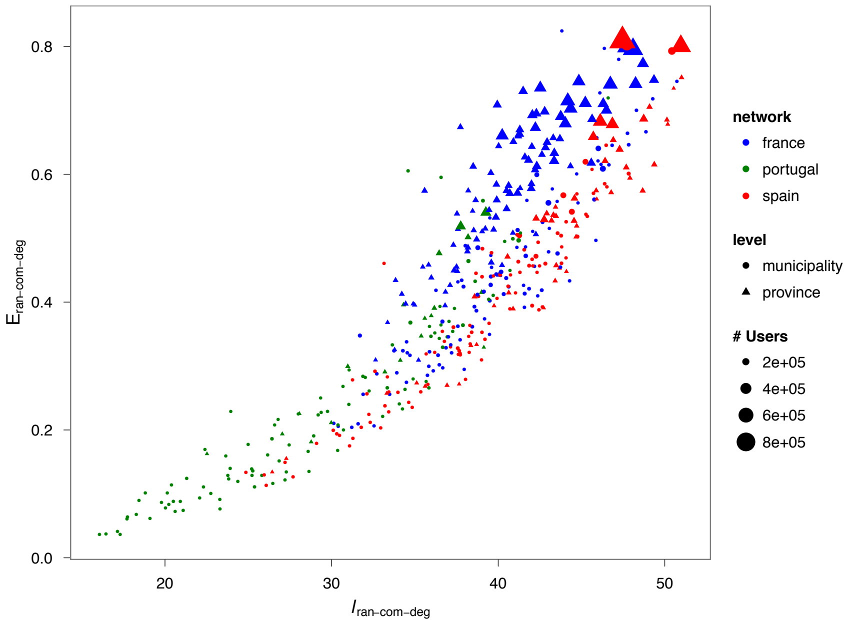

Fig. S16: Correlation between the average shortest path length and the error rate for each province and municipality, using the *ran-com-deg* routing strategy. The size of the symbols is correlated with the corresponding population.

with previous theoretical results: is not feasible to route efficiently in networks with an average degree smaller than 4. In fact as we show in figure S17(bottom), networks with low average degree actually have a significantly larger diameter.

3.3 Relation to decentralized routing theory

As mentioned in the paper, a number of approaches have been employed in the literature to explain the capability of humans participating in Milgram-like experiments to find short paths: repetitions of the experiment asking the participants about routing criteria are performed [11, 26], computer simulation of decentralized search strategies are tested on real network data [22, 2], and analytic studies focusing on certain properties of networks are conducted [37, 19]. In this last category, lots of attention was attracted by Kleinblerg's work [19, 16] where it is proven that a regular two dimensional lattice can obtain small world structure by adding randomly links between nodes. Additionally, only if these links are added with probablity $\frac{1}{r^2}$[8], a decentralized algorithm is able to find these short paths. Even if this is indeed a very interesting finding, we cannot map our phone network on a two dimensional lattice with additional long-range links.

However, in [17, 18] the same author proposes a generalization which we can in fact apply, which is called the *group model*. In short, let be a network whose node set is V, a set of *groups*, $\mathcal{S} = \{S_1, S_2 ... S_n\}$ where $S_i = \{v_1, v_2, ... v_i / v_i \in V\}$ and at least one of the groups S_i is the full vertex set V. Under these asumptions, for any pair of nodes (u, v) a function $g(u, v)$ can be defined such as $g(u, v)$ is the size of the smallest group S_i containing both u and v. If a network is constructed so that k edges are added to each node with probability proportional to $g^{-\gamma}(u, v)$ where $\gamma = 1$, then a decentralized algorithm can route in polylogarithmic time. If the network is constructed with $\gamma < 1$ there is no logarithmic routing and if $\gamma > 1$ there are networks where decentralized routing can be successful.

Both our main routing strategies, communities and geography, can be mapped to groups[9]: it is straightforward in the case of communities since the hierarchy resulting of community detection is a valid set of groups S. For geography, we can consider $g(u, v)$ as the number of people who are closer from v than u, which means S_i are the *balls* of population centered in a tower with a given radius r. A similar model was actually proposed in [22] to explain how a simple *geogreedy* technique is capable of sending messages to the right city.

On a first look both geographically determined balls and communities seem to have the correct exponent as shown for Lisbon in Figure S18. However when we calculate the scailing, we find $\gamma_{geo} = 0.85$ and $\gamma_{com} = 1.07$. When we apply

[8] r denotes the Manhattan distance between two given nodes in the lattice

[9] There are some characteristics in our networks which makes them different from the theoretical model: our networks have heterogenous degree and we need to relax some of the properties of the groups, especially in the case of geographic balls. Concretely, the original model requires that for any group S_i of size $g >= 2$ containing a node v, there has to be a group $S_j \subseteq S_i$ containing v which is strictly smaller than S_i, but contains at least $min(\lambda g, g-1)$ nodes, where $\lambda < 1$. To accomplish this in our case, we need to choose a λ arbitrary small, at most $1/t_{max}$, where t_{max} is the maximum number of users in one tower.

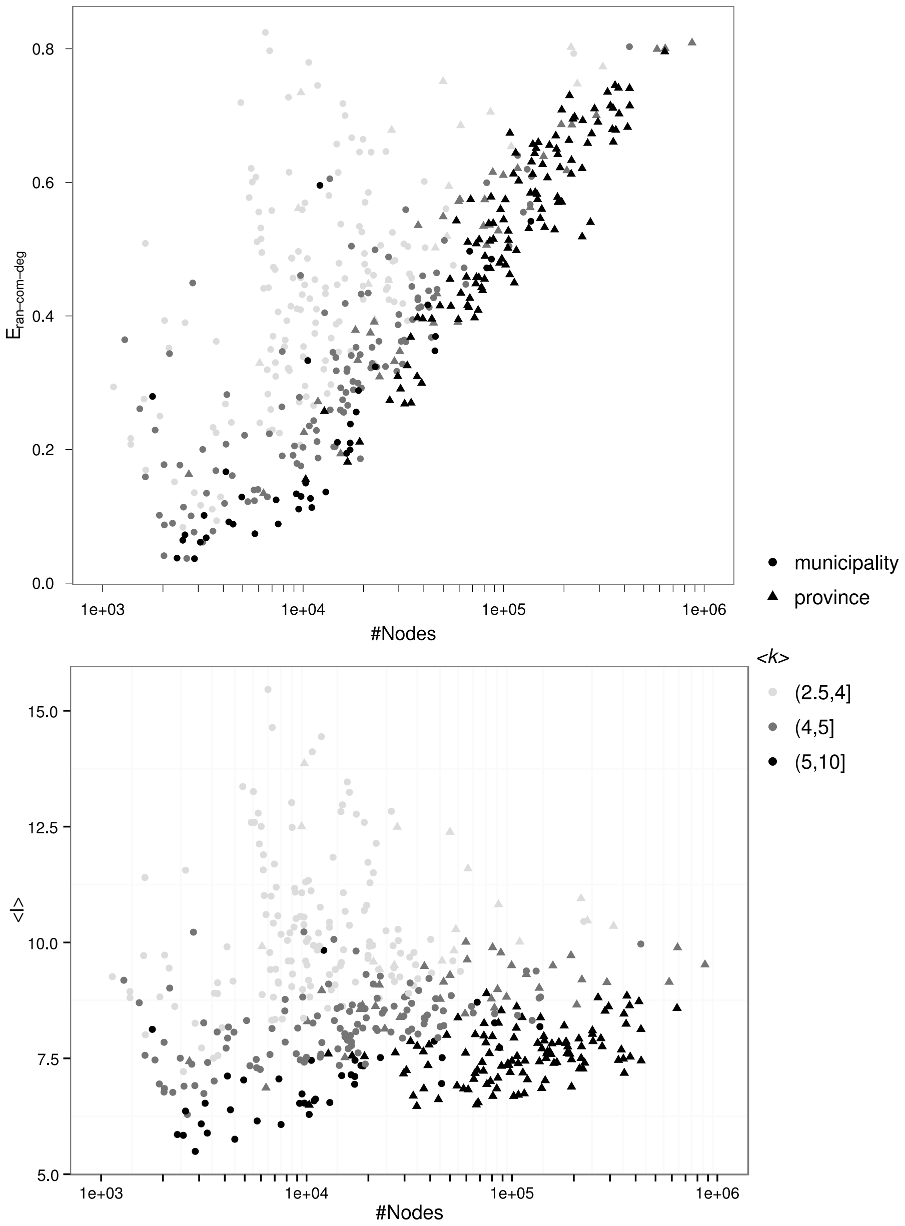

Fig. S17: Scaling of error rate with size for the *ran-com-deg* strategy (top). Colors represent the average degree $\langle k \rangle$. If networks are connected enough $\langle k \rangle > 4$, scaling follows a logarithmic behavior. A similar behavior emerges in the scaling of the average path length $\langle l \rangle$ (bottom), where networks with low degree have a diameter relatively large for their size.

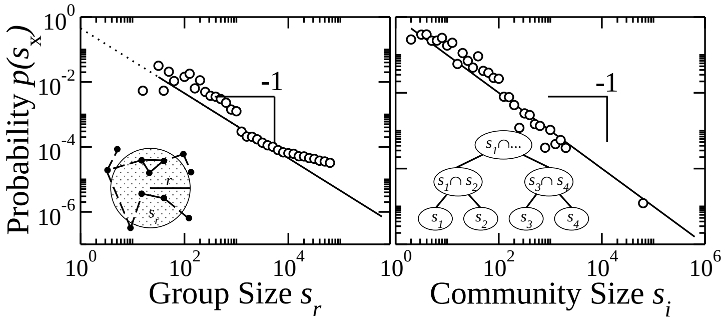

Fig. S18: Probability of two nodes in the Lisbon urban network to be connected if they belong to a geographical or community group of size S. Both distributions are close to the theoretical S^{-1} needed for networks to be searchable with a decentralized algorithm

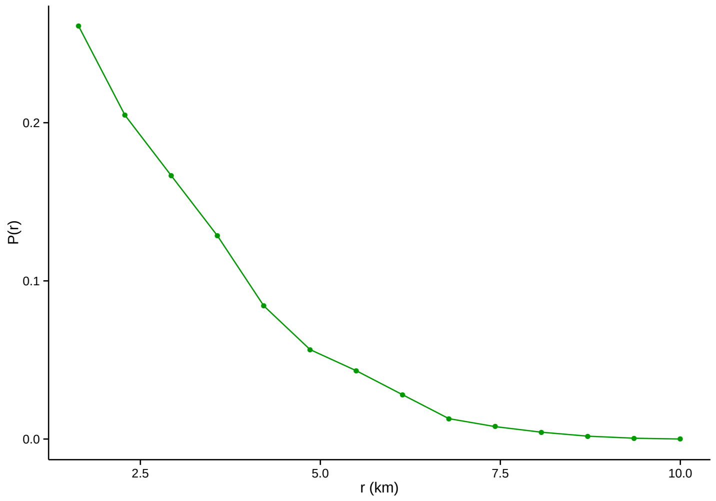

Fig. S19: Probability of finding a link within distance r in the Porto urban network. Despite the lack of spatial correlation found in urban communities (see figure 6 in the manuscript), social ties are still noticeably influenced by physical distance.

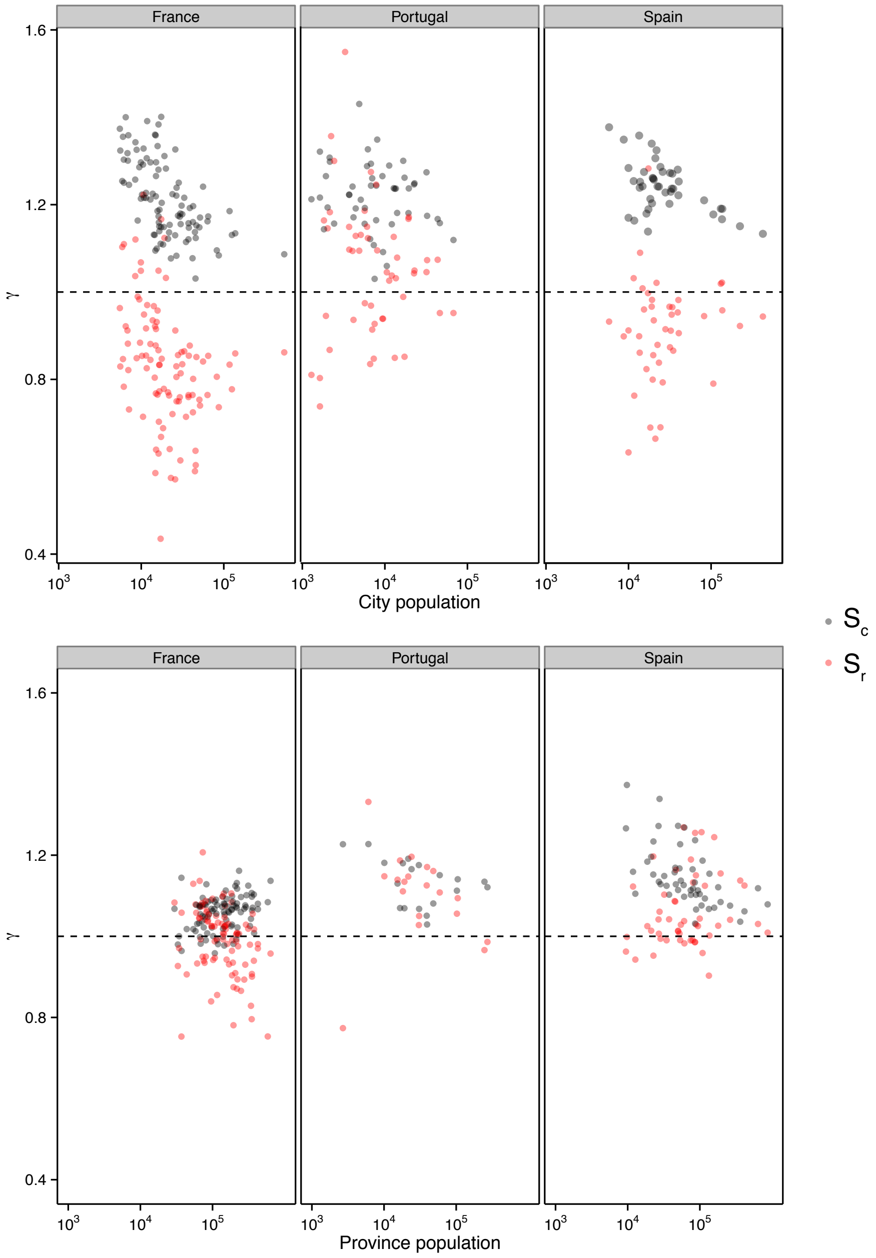

Fig. S20: Different γ values obtained for geographical and communities groups in all provinces and cities in each country. Results confirm theoretical predictions since in those scenarios where *geo* is not efficient (i.e. cities), $\gamma_{geo} < 1$ while communities show the correct behaviour even within cities. Note some municipalities in Portugal have the right γ_{geo}, which is easy to understand when we explore them and find in rural areas in Portugal, municipalities are actually a set of towns so geographic routing is still efficient to some point because it can find the right town.

this fitting procedure to all cities and provinces in the 3 countries, we find γ_{geo} consistently bellow one and $\gamma_{com} > 1$ for cities while we observe no significant difference on the province level (see Figure S20).

The explanation relies on the following fact: given a group S where the target belongs (can be a geographic ball or a community), a decentralized algorithm tends to search the whole group before trying nodes in other groups. If nodes in the group do not form a giant connected component on the network, there are no paths between most of node pairs $u, v \in S$ where all the nodes on the path are also in S. In this case, the decentralized search fails. In figure 2d of the paper we show the difference between geographic balls and communities: while communities are by definition connected, geographic balls lose connectivity for small radius. This means, within the same tower, there are *islands* of users. However, as we can see in the figure, if we calculate the giant components of the geographic balls on the country scales (locating users in municipalities) we observe no such breakdown. This finding agrees with the fact that *geo* strategies are actually efficient on the country scale, as discussed in the previous section.

3.4 Connectivity collapse within cities

As we have discussed in the previous section, given a ball of radius r km, if we construct the social network between the people living in municipalities within the ball, this network will have a giant component (figure 4b in the paper). However, if we choose a ball within a municipality, and build the network between people living within the same towers, the giant component vanishes. In figure S21 we show the reason for this collapse, by studying the intra-tower networks for the 30 top towers in each capital city and then compare to two randomized versions of the networks. The first randomization keeps average degree (Erdös-Rényi), and the second keeps the whole degree distribution, but both eliminate clustering. Our results demonstrate that clustering is the main responsible for the absence of a giant component.

In summary we have strong evidence that the observed relation between geographic space and social network (connected pieces of land produce connected networks) breaks within cities. Thus, we neither can find a distance $r_{critical}$ nor a geographical group size $S_{critical}$ below which there is no connected component in the induced subgraph, because cities have very different extension and population. To support this claim we have studied all intra-tower networks in the capital cities and compared to municipalities networks of the same size, and results are presented in Figure 5 in the manuscript.

Number of social ties within towers

Our results in figure 2 in the paper agree with previous literature [21, 22] finding that the probability of two users within distance r to be connected decreases similar to $\frac{1}{r}$. However, this finding does not give us any guideline about the number of links between people within the same tower, since in principle they are within $r = 0$ distance. In order to be able to apply pure geographical models

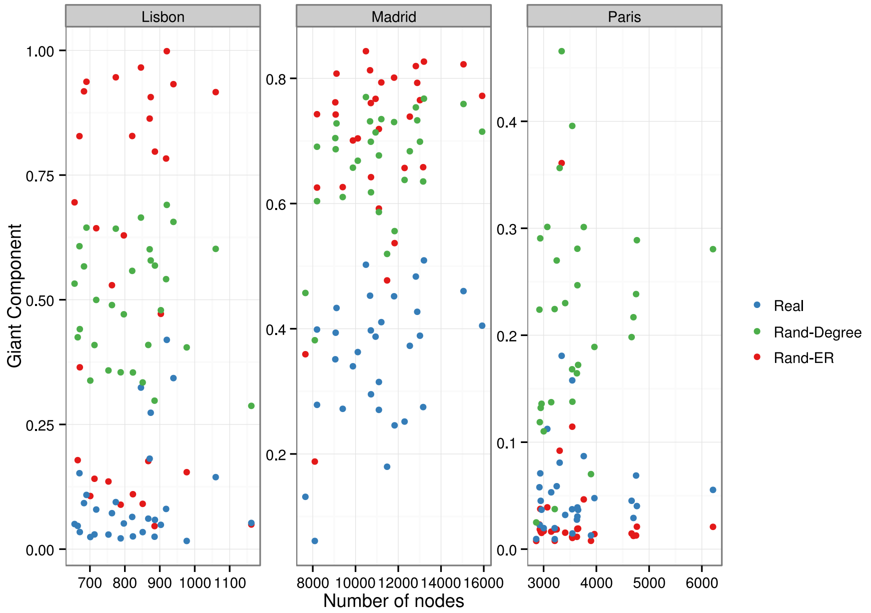

Fig. S21: For each of the top 30 towers in each capital city, the fraction of nodes in the giant component is computed. Additionally, the giant components for randomized versions Rand-ER (keeps average degree) and Rand-Degree (keeps degree distribution) are shown. Each random point in the graph is averaged over hundred realizations of the randomization process.

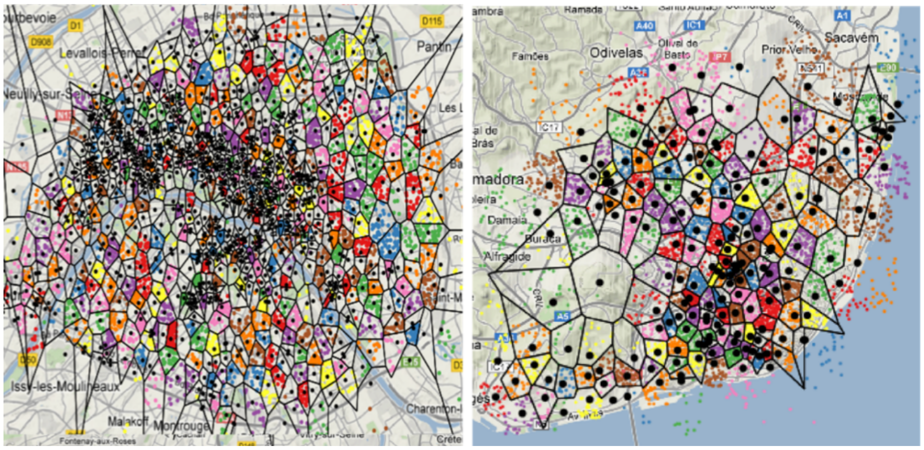

Fig. S22: Randomization of user location within their own Voronoi cell for Lisbon and Paris. Figures displays the given locations for two thousand random users in the city. Our randomization keeps spatial distribution in the tower level (that is why small downtown cells appear to be full). The maps were created using the R packages *ggmap* and *ggplot2*.

to our data, we have to randomize the position of the users around the tower's location.

A common assumption for mobile phone data is considering that if a call is processed by a tower, then that tower is the closest to the user's location. This assumption implies the geographic space can be divided according to the Voronoi diagram of the towers in that region. This way our randomization assigns a users a position uniformly distributed in the Voronoi cell they belong. Figure S22 shows the randomization process in Paris and Lisbon[10].

3.5 Crossover in geography-based routing

Figure S24 shows the performance of different routing strategies in the intracity scenario considering that a delivery is succesful if the message was able to reach the target in less than 50 steps (figure 3b in the paper is analogous to this figure but with 100 steps threshold). One interesting aspect is the crossover behavior between municipality and provinces in the geographic based routing. In this section we explain the emergence of such behavior by using a simplified example.

The crossover can not be linked to a critical spatial characteristic of the city. As shown in Fig. S23, we do not find a critical city diameter, area, or density below which the routing fails. This is a strong indication that the geography plays a different role in the social network structure between and within cities.

[10] This simulation could not be computed in Madrid because the Voronoi assumption is not valid for zipcodes.

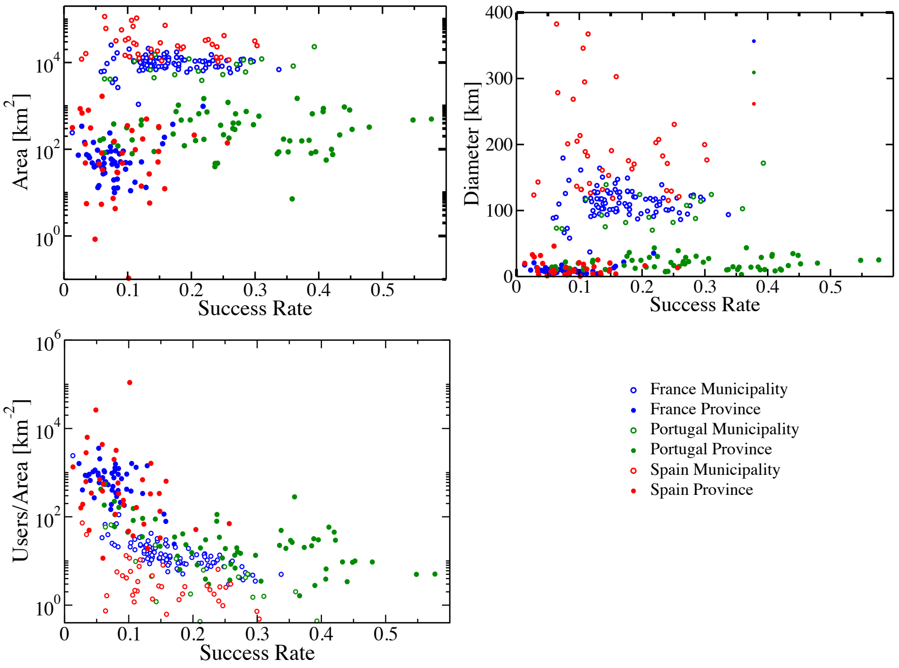

Fig. S23: The success rate does not seems to be highly correlated with spatial characteristics of the studied region like the diameter, the area, and the density of the studied region. Therefore, no critical boundary below which geographical routing fails can be identified.

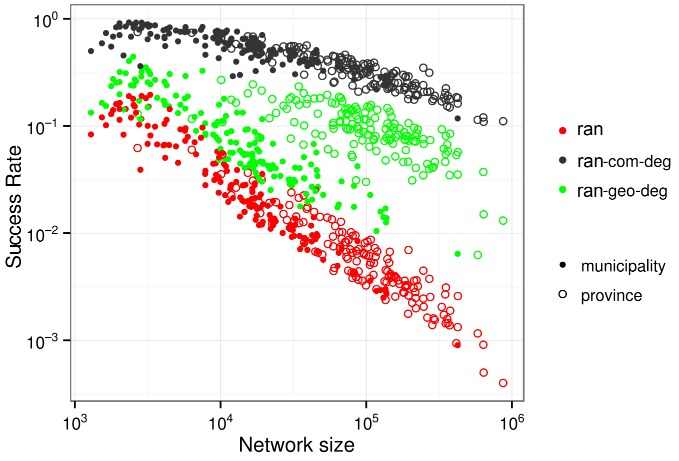

Fig. S24: Success rate for different routing strategies in provinces and municipalities with $\langle k \rangle > 4$. This figure is equivalent to figure 2b in the main paper, but considering successful routing if the message was delivered within 50 steps, instead of 100. Pure *ran* routing produces a reverse linear decrease, while the community based routing produces a much slower decay. Geographical routing in the intracity scenario produces a crossover between behavior provinces and municipalities.

Figure S25 shows a simplified version of a province with N users and 3 cities. Let's denote $P(S)$ the probability that a message is succesfully delivered. For *ran* algorithm it is straightforward to conclude the probability $P_{ran}(S) = 1/N$ being N the number of nodes in the network, no matter if the network represents a province or a city. This conclusion agrees with our results in figures S24 and 3b in the paper.

However, for geographic routing, we denote $P(c)$ where $c \in \{A, B, C\}$ the probability of reaching the right city c and $P(S|c)$ being the probability that the message is succesfully delivered given it is already in the right city c. In the intercity experiment scheme (see section 2) we have proven that the *geo* approach is valid, delivering the vast majority of the messages to the right city, so we consider $P_{geo}(c) = 1$[11]. Using results from our intracity experiment we assume $P_{geo}(S|c) = \frac{1}{n_c^\alpha}$, with $0 < \alpha < 1$. Then

$$
\begin{aligned}
P_{geo}(S) &= \sum_{c\in\{A,B,C\}} \frac{n_c}{N} P_{geo}(c) P_{geo}(S|c) = \\
&= \sum_{c\in\{A,B,C\}} \frac{n_c}{N}\frac{1}{n_c^\alpha} = \frac{\sum_{c\in\{A,B,C\}} n_c^{(1-\alpha)}}{N} \geq \frac{(\sum_c n_c)^{(1-\alpha)}}{N} = \frac{1}{N^\alpha},
\end{aligned}
$$

which means that using *geo* approach, a province with a certain population N has a higher success rate than a municipality with the same size. Even if we generalize $P_{geo}(S|c) = f(n_c)$ where f is any decreasing function this result holds: if *geo* is capable to deliver all messages to the right city, then $P_{geo}(S)$ is a weighted average of the performances in the cities forming the province such that $f(n_{max}) \leq P_{geo}(S) \leq f(n_{min})$ where n_{min} and n_{max} denote the size of the smallest and biggest cities respectively.

References

[1] Dimitris Achlioptas, Raissa M D'Souza, and Joel Spencer. Explosive percolation in random networks. *Science*, 323(5920):1453–1455, 2009.

[2] L. Adamic and E. Adar. How to search a social network. *Social Networks*, 27(3):187–203, 2005.

[3] Yong-Yeol Ahn, James P Bagrow, and Sune Lehmann. Link communities reveal multiscale complexity in networks. *Nature*, 466(7307):761–764, 2010.

[4] Nuno AM Araujo and Hans J Herrmann. Explosive percolation via control of the largest cluster. *Physical Review Letters*, 105(3):35701, 2010.

[5] V. Blondel, G. Krings, and I. Thomas. Regions and borders of mobile telephony in belgium and in the brussels metropolitan zone. *Brussels Studies*, 42(4), 2010.

[11] This is a fair assumption since experimental results found error rates $E < 10^{-3}$

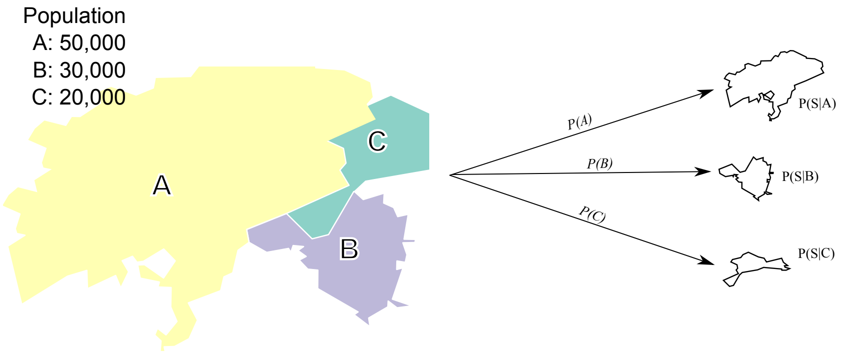

Fig. S25: Simplified version of a province with population $N = 10^5$ and 3 municipalities. Routing process can be divided into 2 steps: reaching the right municipality and then finding the right target within that city. $P(A)$ denotes the probability that a message whose target is in city A actually reaches A. $P(S|A)$ denotes the probability that a message reaches its target given it is already in A. *Geo* strategy is efficient to reach the right city so $P(A) = P(B) = P(C) = 1$ which implies the performance on the overall province is actually better than in the major city, producing the crossover observed in the results. The figure was created using the R package *maptools*.

[6] V.D. Blondel, J.L. Guillaume, R. Lambiotte, and E. Lefebvre. Fast unfolding of communities in large networks. *Journal of Statistical Mechanics: Theory and Experiment*, 2008(10):P10008, 2008.

[7] Bela Bollobás and Fan R. K. Chung. The diameter of a cycle plus a random matching. *SIAM Journal on discrete mathematics*, 1(3):328–333, 1988.

[8] F. Calabrese, D. Dahlem, A. Gerber, D. Paul, X. Chen, J. Rowland, C. Rath, and C. Ratti. The connected states of america: Quantifying social radii of influence. In *Privacy, security, risk and trust (passat), 2011 ieee third international conference on and 2011 ieee third international conference on social computing (socialcom)*, pages 223–230. IEEE, 2011.

[9] E. Cho, S.A. Myers, and J. Leskovec. Friendship and mobility: user movement in location-based social networks. In *Proceedings of the 17th ACM SIGKDD international conference on Knowledge discovery and data mining*, pages 1082–1090. ACM, 2011.

[10] Aaron Clauset, Cosma Rohilla Shalizi, and Mark EJ Newman. Power-law distributions in empirical data. *SIAM review*, 51(4):661–703, 2009.

[11] Peter Sheridan Dodds, Roby Muhamad, and Duncan J Watts. An experimental study of search in global social networks. *Science*, 301(5634):827–829, 2003.

[12] Robin IM Dunbar. Neocortex size as a constraint on group size in primates. *Journal of Human Evolution*, 22(6):469–493, 1992.

[13] P. Expert, T.S. Evans, V.D. Blondel, and R. Lambiotte. Uncovering space-independent communities in spatial networks. *Proceedings of the National Academy of Sciences*, 108(19):7663–7668, 2011.

[14] Santo Fortunato. Community detection in graphs. *Physics Reports*, 486(3):75–174, 2010.

[15] B. Goncalves, N. Perra, and A. Vespignani. Modeling users' activity on twitter networks: validation of dunbar's number. *PLoS One*, 6(8):e22656, 2011.

[16] Jon Kleinberg. The small-world phenomenon: an algorithm perspective. In *Proceedings of the thirty-second annual ACM symposium on Theory of computing*, pages 163–170. ACM, 2000.

[17] Jon Kleinberg. Complex networks and decentralized search algorithms. In *Proceedings oh the International Congress of Mathematicians: Madrid, August 22-30, 2006: invited lectures*, pages 1019–1044, 2006.

[18] Jon Kleinberg et al. Small-world phenomena and the dynamics of information. *Advances in neural information processing systems*, 1:431–438, 2002.

[19] Jon M Kleinberg. Navigation in a small world. *Nature*, 406(6798):845–845, 2000.

[20] G. Krings, F. Calabrese, C. Ratti, and V.D. Blondel. Urban gravity: a model for inter-city telecommunication flows. *Journal of Statistical Mechanics: Theory and Experiment*, 2009(07):L07003, 2009.

[21] R. Lambiotte, V.D. Blondel, C. De Kerchove, E. Huens, C. Prieur, Z. Smoreda, and P. Van Dooren. Geographical dispersal of mobile communication networks. *Physica A: Statistical Mechanics and its Applications*, 387(21):5317–5325, 2008.

[22] D. Liben-Nowell, J. Novak, R. Kumar, P. Raghavan, and A. Tomkins. Geographic routing in social networks. *Proceedings of the National Academy of Sciences of the United States of America*, 102(33):11623–11628, 2005.

[23] Christopher McCarty, Peter D Killworth, H Russell Bernard, Eugene C Johnsen, and Gene A Shelley. Comparing two methods for estimating network size. *Human Organization*, 60(1):28–39, 2001.

[24] Tyler H McCormick, Matthew J Salganik, and Tian Zheng. How many people do you know?: Efficiently estimating personal network size. *Journal of the American Statistical Association*, 105(489):59–70, 2010.

[25] A. Mislove, M. Marcon, K.P. Gummadi, P. Druschel, and B. Bhattacharjee. Measurement and analysis of online social networks. In *Proceedings of the 7th ACM SIGCOMM conference on Internet measurement*, pages 29–42. ACM, 2007.

[26] R. Muhamad. *Search in Social Networks*. PhD thesis, Columbia University, 2010.

[27] Mark EJ Newman. Modularity and community structure in networks. *Proceedings of the National Academy of Sciences*, 103(23):8577–8582, 2006.

[28] Mark EJ Newman. Random graphs with clustering. *Physical Review Letters*, 103(5):58701, 2009.

[29] J.P. Onnela, S. Arbesman, M.C. González, A.L. Barabási, and N.A. Christakis. Geographic constraints on social network groups. *PLoS One*, 6(4):e16939, 2011.

[30] J.P. Onnela, J. Saramäki, J. Hyvönen, G. Szabó, D. Lazer, K. Kaski, J. Kertész, and A.L. Barabási. Structure and tie strengths in mobile communication networks. *Proceedings of the National Academy of Sciences*, 104(18):7332–7336, 2007.

[31] G. Palla, A.L. Barabasi, and T. Vicsek. Quantifying social group evolution. *Nature*, 446(7136):664–667, 2007.

[32] C. Ratti, S. Sobolevsky, F. Calabrese, C. Andris, J. Reades, M. Martino, R. Claxton, and S.H. Strogatz. Redrawing the map of great britain from a network of human interactions. *PLoS One*, 5(12):e14248, 2010.

[33] S. Scellato, A. Noulas, and C. Mascolo. Exploiting place features in link prediction on location-based social networks. In *Proceedings of the 17th ACM SIGKDD international conference on Knowledge discovery and data mining*, pages 1046–1054. ACM, 2011.

[34] F. Simini, M.C. González, A. Maritan, and A.L. Barabási. A universal model for mobility and migration patterns. *Nature*, 484(7392):96–100, 2012.

[35] Alessandro Vespignani. Modelling dynamical processes in complex socio-technical systems. *Nature Physics*, 8(1):32–39, 2011.

[36] Duncan Watts and S Strogatz. The small world problem. *Collective Dynamics of Small-World Networks*, 393:440–442, 1998.

[37] Duncan J Watts, Peter Sheridan Dodds, and Mark EJ Newman. Identity and search in social networks. *science*, 296(5571):1302–1305, 2002.
